# Cube-shaped Cobalt-doped zinc oxide nanoparticles with increased visible-light-driven photocatalytic activity achieved by green co-precipitation synthesis

**DOI:** 10.1038/s41598-023-46464-7

**Published:** 2023-11-07

**Authors:** Asmaa I. Meky, Mohamed A. Hassaan, Howida A. Fetouh, Amel M. Ismail, Ahmed El Nemr

**Affiliations:** 1 Department of Chemistry, Faculty of Science, Alexandria University, Alexandria, Egypt; 2 Environment Division, National Institute of Oceanography and Fisheries (NIOF), Kayet Bey, Elanfoushy, Alexandria, Egypt

**Keywords:** Pollution remediation, Environmental sciences, Environmental chemistry

## Abstract

From the perspective of environmental protection, the highly efficient degradation of antibiotics and organic dyes in wastewater needs to be tackled as soon as possible. In this study, an ecofriendly and green cube-shaped cobalt-doped zinc oxide nanoparticles (Co–ZnO NPs) photocatalyst using *Pterocladia Capillacea (P. Capillacea)* water extract loaded with 5, 10, and 15% cobalt ions were formed via co-precipitation process to degrade antibiotics. The prepared Co–ZnO NPs were tested as a photocatalyst for the photodegradation of ciprofloxacin (CIPF) in the presence of a visible LED-light source. Co–ZnO NPs have been obtained through the co-precipitation method in the presence of *P. Capillacea* extract as a green capping agent and reducing agent, for the first time. Several characterization techniques including FTIR, XRD, BET, XPS, TEM, EDX, SEM, TGA and DRS UV–Vis spectroscopy were applied to study the prepared Co–ZnO NPs. XRD results suggested that the average size of these NPs ranged between 42.82 and 46.02 nm with a hexagonal wurtzite structure. Tauc plot shows that the optical energy bandgap of ZnO NPs (3.19 eV) gradually decreases to 2.92 eV by Co doping. Examinations showed that 5% Co–ZnO NPs was the highest efficient catalyst for the CIPF photodegradation when compared with ZnO NPs and other 10 and 15% Co–ZnO NPs. A 10 mg/L solution of CIPF was photo-degraded (100%) within the first 15 min irradiation. The kinetics showed that the first-order model is suitable for displaying the rate of reaction and amount of CIPF elimination with *R*^2^ = 0.952. Moreover, central composite design optimization of the 5% Co-doped ZnO NPs was also investigated.

## Introduction

Antibiotics are widely used medications that are challenging to remove from wastewater using traditional water treatment techniques like coagulation or filtration^1–4^. The second-generation quinolone antibiotic ciprofloxacin (CIPF) is one of the most often used antibiotics in the world due to its broad-spectrum antibacterial action and resistance to a variety of infections. However, total metabolic breakdown of CIPF was not possible. Over 75% of CIPF is eliminated from the living organism in an unmetabolized form, where it eventually enters municipal wastewater^5^. Cosmetic, textile, food and plastic industries are the main sources of producing organic dyes containing wastewater. A family of contaminations are seriously harmful to biota, plants, and human even in negligible concentrations since its degradation, in particular photodegradation, produce invasive species, radicals, in aquatic medium^6^. Photocatalytic degradation has been confirmed to be a promising removal method to lessen the hazards due to the presence of organic dyes in the environment^7–9^.

Photocatalysis has been attracting increasing attention due to its great potential in solving global challenging problems, e.g. the release of harmful contaminants into the ecosystem, energy-based concerns, global warming arising from the increase of greenhouse gases in the atmosphere, etc.^6–12^. It is of great significance to find reliable methods for the removal of a large variety of environmental pollutions in surface water taking advantage of the natural energy source available, solar light^13^. Semiconductor photocatalysis (such as BiOBr, TiO_2_, WO_3_, SrTiO_3_, Ag_2_MoO_4_ and ZnO) is regarded as an effective way to solve air pollution problems. As an advanced oxidation technology, it can degrade organic pollutants into H_2_O and CO_2_ by using the coupling of light and semiconductors to produce active species with redox capabilities^13^. However, the majority of them behave with inferior light absorption properties, which restricts their application^13,14^.

The possibility of treating wastewater using several nanomaterials, including copper oxide^14^, titanium dioxide^15^, magnesium-doped zinc oxide^16^, silver-doped titanium dioxide^17^, and iron^18^, has been examined. Zinc oxide (ZnO) is one of the most extensively studied semiconductors with a large band gap of about 3.3 eV. It's known to have a stable hexagonal wurtzite structure and a high excitation binding energy of roughly 60 meV at ambient temperature^19^.

When the ZnO-NPs semiconductor is exposed to UV light, the electrons in its valence band move to the conduction band and produce a hole-electron (h/e) pair, which leads to the generation of hydroxyl radicals and reactive oxygen species (ROS)^20^. According to several studies, ZnO is a better photocatalyst than TiO_2_^16,21^. ZnO has thus been chosen as the photocatalyst in this investigation due to its high catalytic efficiency, low cost, and non-toxic makeup^16^. Various methods, such as the study of metal or nonmetal-doped ZnO NPs, were investigated to create ZnO NPs suitable for photocatalysis by solar light^22–26^. It is known that metal ions doping influences semiconductor materials' capacity to absorb light, as well as promoting interfacial charge transfer reactions and boosting their photocatalytic properties^27^. It was successful in employing cobalt as a dopant metal to improve the electrical and optical properties of the ZnO lattice and to induce a significant redshift in band gap energy, which makes it easier to transport electrons and tune the ZnO Fermi level^22^.

Highly stable nanoparticles are produced using an ecofriendly synthesis method^28,29^. Green synthesizing pathways operate in a simple, affordable, non-toxic, and environmentally friendly^30,31^. Microalgae and seaweed are aquatic plants that may grow on surfaces that are typically inappropriate for other applications, produce the greatest biomass, have the best photosynthetic efficiency, and are resistant to several pollutants^32–40^. Seaweeds have significant metal-binding capacities due to the presence of proteins, polysaccharides, and lipids with diverse functional groups on the surface of their cell walls^28^. In earlier works, Hamidian et al.^30^ presented an environmentally friendly approach for the synthesis of ZnO and 1, 3, 7, and 10% Co-doped ZnO nanowires by using *Salvadora persica* aqueous extract. The photocatalytic activity of the generated samples indicated that doped cobalt in ZnO has finally reached its peak in terms of speeding up photocatalyst performance. The ideal sample had 20 mg/L of 1% Co–ZnO nanowires, which removed 89% of the MB dye. Zelekew et al.^41^ synthesized cobalt-doped ZnO by accumulating cobalt ions on *Eichhornia crassipes* plant tissue for various lengths of time and combining them with zinc precursor. ZnO NPs were created by Mansour et al.^28^ using red seaweed (*Pterocladia capillacea*) and they were tested for their ability to adsorb Ismate Violet 2R (IV2R) dye ions from water solution. By degrading the color Malachite Green while being exposed to UV light, ZnO NPs' photocatalytic activity was examined. *Pomegranate Punica granatum* fruit peel extract was used as the stabilizing agent during the biosynthesis of the ZnO NPs^12^. In this research, we tried to use green chemistry methods to prepare Co–ZnO Nps in the presence of *P. Capillacea* extract as a capping agent and reducing agent. The Nps produced by the green method have advantages such as non-toxicity, bio-compatibility, very short extraction time, lack of organic solvents, low cost, ease of use, high precision and recovery due to nanoscale structure and having a high ratio of surface to volume. Algae extract can play a role in the synthesis of Co–ZnO Nps through its potential as a natural source of bioactive compounds. To the authors' knowledge, the synthesis of Co–ZnO NPs photocatalyst using *P. Capillacea* extract is used for the first time in CIPF.

A potent method known as response surface methodology (RSM) can forecast the interactions between independent parameters and system responses. RSM has not yet been used to investigate the process factors involved in the seed-mediated synthesis of ZnO NPs. In conclusion, this ground-breaking study investigates the optimization of operational factors for the effective biosynthesis of ZnO NPs using RSM^42^. This study aims to afford a deep insight into the photocatalytic degradation of ciprofloxacin as a fluoroquinolone antibiotic and fully understand the origin of the role of Co–ZnO NPs in the photocatalytic process with different concentrations (5, 10 and 15%) of cobalt, estimate the role of Co–ZnO NPs in antibiotic degradation and elucidate the CIPF degradation pathways.

## Materials and methods

### Chemicals and equipment

Zinc acetate dihydrate, cobalt acetate tetrahydrate, NaOH, isopropanol (IPA), Na-EDTA (Ethylenediaminetetraacetic acid) and Benzoquinone (BQ) were purchased from Sigma Aldrich, USA. Ciprofloxacin (CIPF) was purchased as a Ciprofloxacin 200 mg/100 mL I.V. infusion solution from Amirya Pharmaceuticals, Alexandria, Egypt. Fresh red algal biomass *P. capillacea* was collected from Abo-Quir Bay, Alexandria, Egypt. The following instruments were applied to identify the samples of ZnO NPs and Co–ZnO NPs photocatalysts. ZnO and Co–ZnO NPs crystallinity and average crystal size were confirmed by Bruker Meas Srv XRD (D2-diffractometer that controls at 30 kV, 10 mA using Cu tube of λ = 1.5418 Å and 2θ with a temperature range of 5–80°) was used. Fourier transform infrared (FTIR) analysis was made using a VERTEX70 instrument linked to Platinum ATR model V-100, Bruker, Germany, in the wavenumber range of 400–4000 cm^−1^. Scanning electron microscope (SEM) (SEM- JEOL, IT 200 Japan) equipped with Energy dispersive X-ray spectroscopy (EDX) was used to determine the elemental analysis, materials' morphology and surface characteristics. A transmission electron microscope (TEM) (JTM 1400 plus-Japan) was applied to define the size and shape of the nanostructures. UV–Visible (UV–Vis), GBC Cintra 3030 at the range 190–900 nm spectrophotometer was applied to measure the optical absorbance of these samples. Using the BELSORP—Mini II from BEL Japan, Inc., the mean pore diameter and specific surface area (BET, Brunauer Emmett-Teller) were measured. The SDT650-Simultaneous Thermal analyzer equipment was used to conduct thermal analyses of samples utilizing a 10 °C per minute ramping temperature. Monochromatic X-ray Al K-alpha radiation with a spot size of 400 µm and a pressure of 10^–9^ mbar was used to gather X-ray photoelectron spectroscopy (XPS) data on K-ALPHA (Thermo Fisher Scientific, USA) with full spectrum pass energy of 200 eV and a narrow spectrum of 50 eV.

### Synthesis of algae and extract

*P. capillacea* species' fresh red algal biomass was gathered from Abu Qir Bay, Alexandria, Egypt. It was washed with distilled water after being washed with seawater, and tap water, and then dried. Following this, the clean algal biomass was dried at 105 °C for 72 h after being sun-dried for two days, and the dried red algae (DRA) was milled for use. A 10 g of the produced DRA powder was added to 250 mL of distilled water. The mixture was stirred at 75 °C for 2 h on a hot plate and then filtered with filter paper. The *P. capillacea* extract was kept at − 20 °C for supplementary processing^43^.

### Synthesis of ZnO and Co-doped ZnO (Zn_1-x_Co_x_O)

Zn(CH_3_COO)_2_.2H_2_O (0.5M) (solution-A) was added to 90 mL of distilled water and followed by dropwise addition of 10 mL of *P. capillacea* extract under continuous stirring at 60 °C for 30 min. NaOH (0.5 M) was added dropwise until the desired pH was reached and a pale white color appeared. The solution was stirred vigorously for 2 h at the same temperature. The resulting white precipitate was then divided, filtered, rinsed repeatedly with distilled water and EtOH, and allowed to dry at 50 °C overnight. The resultant powder was calcined for three hours at 500 °C^44^. The pale white powder of ZnO NPs was carefully collected and kept until use. The cobalt doping ZnO (Zn_1−*x*_Co_*x*_O) NPs were synthesized via the co-precipitation method in the same way. Where different ratios (5, 10, 15% of Co/ZnO) were synthesized by dissolving the desired amount of Co(CH_3_COO)_2_·4H_2_O (solution-B). Then (solution-B) was added dropwise to (solution-A) followed by the same procedures of ZnO NPs synthesis procedures^45^.

### Photocatalytic activity

To determine the best catalyst performance, A specific amount of 100 mg of ZnO and 5, 10, and 15% of Co-doped ZnO NPs were added to a Pyrex glass beaker containing 100 ml of ciprofloxacin with concentrations of 30 ppm at neutral pH for 2 h under (150 W LED-light lamp) as visible LED-light source and the removal efficiency was measured. For the decomposition of CIPF, a 150 W LED light source was used in the photoreactor. In a standard photocatalytic degradation test, the CIPF solution was combined with the photocatalyst in a 100 mL flask beaker, and the mixture was then kept in the dark for 30 min to establish the adsorption–desorption equilibrium. Then, visible light was shone on the reaction medium containing the photocatalyst and CIPF solution. By taking 2 mL of the aliquot CIPF solution at regular time intervals. A UV–Vis spectrophotometer (model Pg/T80 UV/ Vis) was used for CIPF concentration analysis at a wavelength of *λ*_max_ = 270 nm after it had been centrifuged at 6,000 rpm for 30 min. The degradation efficiency was calculated using Eq. (1).

1 $$ Degradation\;efficiency = \frac{{C_{0} - C_{t} }}{{C_{0} }} \times 100 $$

*C*_0_ stands for the CIPF initial concentration, and *C*_t_ refers to the CIPF final concentration in the solution at specific time intervals under irradiation. The optimization of parameters for photodegradation namely, pH, catalyst dosages, CIPF concentrations, temperature and shaking speed were performed to find out the best conditions for the efficient photocatalytic degradation.

### Radical scavenger

To quench photo-generated species (holes (h^+^), hydroxyl radicals ($^{ \cdot } {\text{OH}}$), and superoxide radicals ($^{ \cdot } {\text{O}}_{{2}}^{-}$), which are responsible for catalytic degradation, three scavengers (10 mM Na-EDTA, 1 mM IPA, and 1 mM BQ) were separately added to a 100 mL solution of 30 ppm CIPF initial concentration^46^.

### Experimental design and data analysis

For experimental design, modeling, and optimization, the response surface methodology (RSM), based on central composite design (CCD), is extensively used. In this investigation, CCD statistical software design expert version 13.0.5.0 was employed to optimize several operational parameters for the degradation of CIPF by 5% Co–ZnO NPs under visible light. In this study using the CCD design, a total of 30 runs were considered at 5 levels and 6 replications at the center point (including 16 factorial points and 8 axial points (a = 2) (Table 1). The CIPF degradation rate is the dependent variable, along with other factors including pH, shaking speed, catalyst dosage, and antibiotic dosage. After the experiment's design was finished, the CIPF conducted photodegradation studies to create a suitable model. Quadratic models were used to analyze the acquired data^47^. Equation (2) illustrates the quadratic models' generic form.

2 $$ \eta = b_{0} + \mathop \sum \limits_{i = 1}^{n} b_{i} x_{i} + \mathop \sum \limits_{i = 1}^{n} b_{ii} x_{i} 2 + \mathop \sum \limits_{i = 1}^{n - 1} \mathop \sum \limits_{i = 1}^{n} b_{ij} x_{i} x_{j} $$

where* b*_0_ is the coefficient constant, *ƞ* is the predicted response variable, *b*_ii_ is the quadratic coefficient, *b*_i_ is the linear coefficient, *b*_ij_ is the interaction coefficient, and *x*_i_ and *x*_j_ are the coded values of the parameters^47^.

**Table 1:** Low- and high-level values for independent variables.

Factor	Name	Units	Minimum	Maximum	Mean	Std. dev. ±
A	Catalyst dosage	mg	20	100	60.00	18.19
B	Antibiotic dosage	mg/L	10	50	30.00	9.10
C	Shaking time	min	50	250	150.00	45.49
D	pH		3	11	7.00	1.82

## Results and discussion

### FTIR analysis

**Figure 1:**
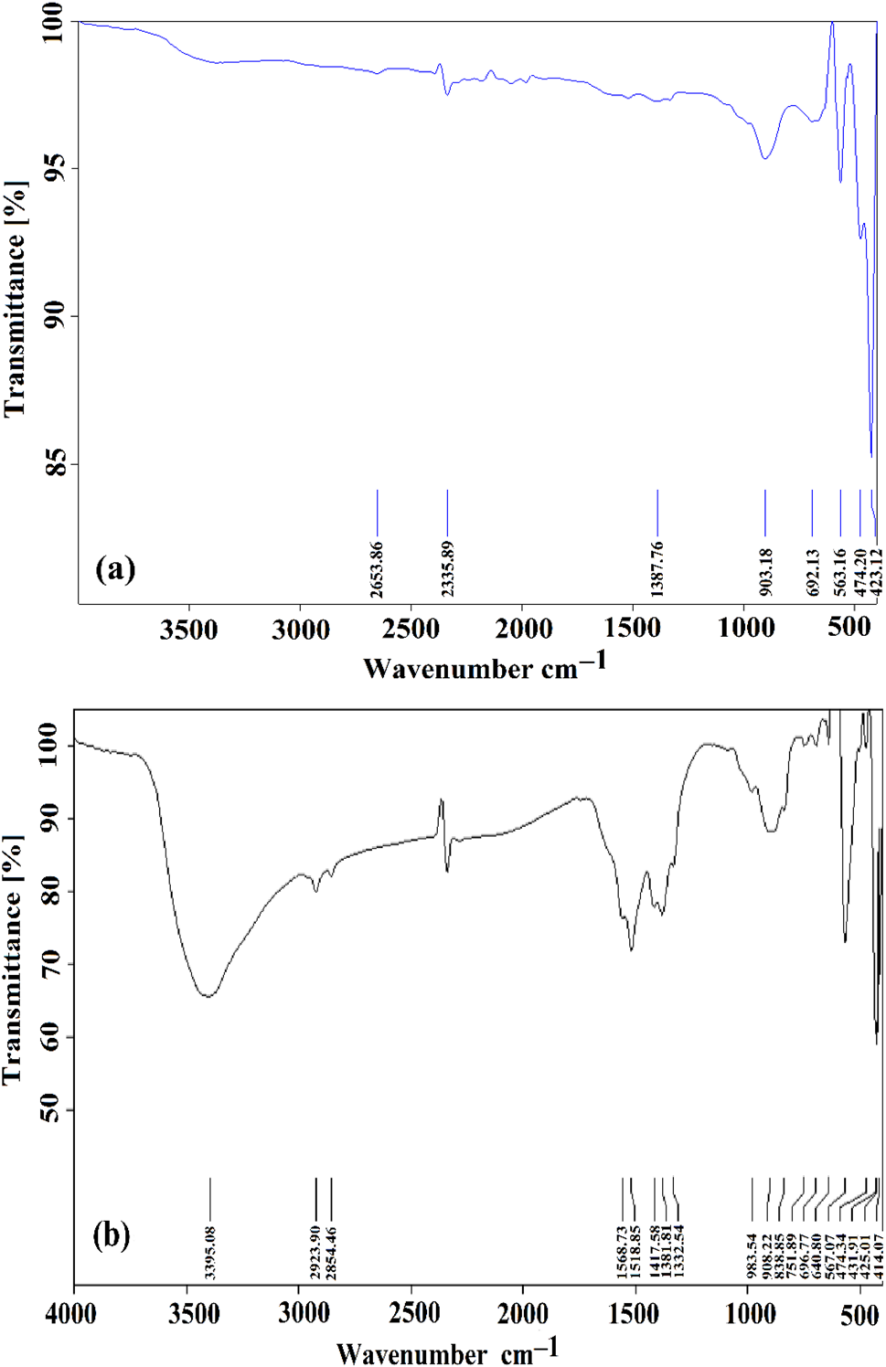
FT-IR bands of (**a**) Green ZnO NPs, and (**b**) Green 5% Co-doped ZnO NPs.

The Fourier-transform infrared (FT-IR) spectrum of Green ZnO NPs was recorded in Fig. 1a in a frequency range of 400–4000 cm^−1^ at room temperature. The peak at 2335 cm^–1^ corresponds to the presence of the O–C–O molecule^48^, and the peak at 2653 cm^–1^ corresponds to the O–H stretch in carboxylic acid^49^. The peaks at 903 cm^–1^ correspond to the internally bonded C–H stretching band. The peak at 1387 cm^–1^ corresponds to the C–C band, while the vibrational stretching at 423–563 cm^–1^ shows the presence of Zn–O corresponding stretching^50^. Figure 1b illustrates how the Co–ZnO NPs sample's FT-IR spectra revealed the sample's obvious functional groups. The Co–ZnO NPs sample demonstrated bands at about 640 cm^−1^ in comparison to ZnO NPs due to the IR absorption of Co–O, further demonstrating the integration of Co^2+^ into the ZnO lattice^48^. The stretching vibration of the Zn–O bonding is recognized to the absorption bands at about 450 cm^−1^^51^. The bands at 1417.56 cm^−1^ during alkane stretching^49^. The peaks at 3395, 1518, and 1558 cm^−1^ showed that water molecules adsorbed on the sample's surface were the cause of the O–H stretching and bending vibrational absorptions. The C=O stretching vibration was visible in the strong bands about 1381 cm^−1^^41^.

### BET analysis

Surface area is crucial for catalytic characteristics since it aids in the adsorption and desorption of reactants. The porosity characteristics of the ZnO, 5, 10, and 15% Co–ZnO nanoparticles were investigated using BET surface area. The N_2_ adsorption/desorption isotherms and the plot of the pore size distribution of ZnO, 5, 10, and 15% Co–ZnO nanoparticles are shown in Fig. S1. The ZnO NPs exhibit a type IV curve (Fig. S1) with a type H3 hysteresis loop, which is classified by the IUPAC and attributed to the prevalence of mesopores^52^. The Green ZnO nanoparticles had a surface area of 5.031 m^2^/g, while the surface areas of the Co–ZnO NPs at 5, 10, and 15% were 15.114 (Table 2), 21.985 (Table S1), and 27.207 m^2^/g (Table S1), respectively.

**Table 2:** Analysis of the surface area of Green ZnO NPs and Green 5% Co–ZnO NPs.

Parameter	Unit	ZnO NPs	5% Co–ZnO NPs
*a*_s,BET_	m^2^∕g	5.031	15.114
*V*_m_	(cm^3^ STP)/g	1.156	3.4726
Mean pore diameter *P*_m_	nm	13.71	14.356
*V*_T_	cm^3^/g	0.01656	0.0542245
*V*_p_	cm^3^/g	0.016727	0.054121
*a*_p_	m^2^/g	5.4328	15.557

### Scanning electron microscope (SEM)

**Figure 2:**
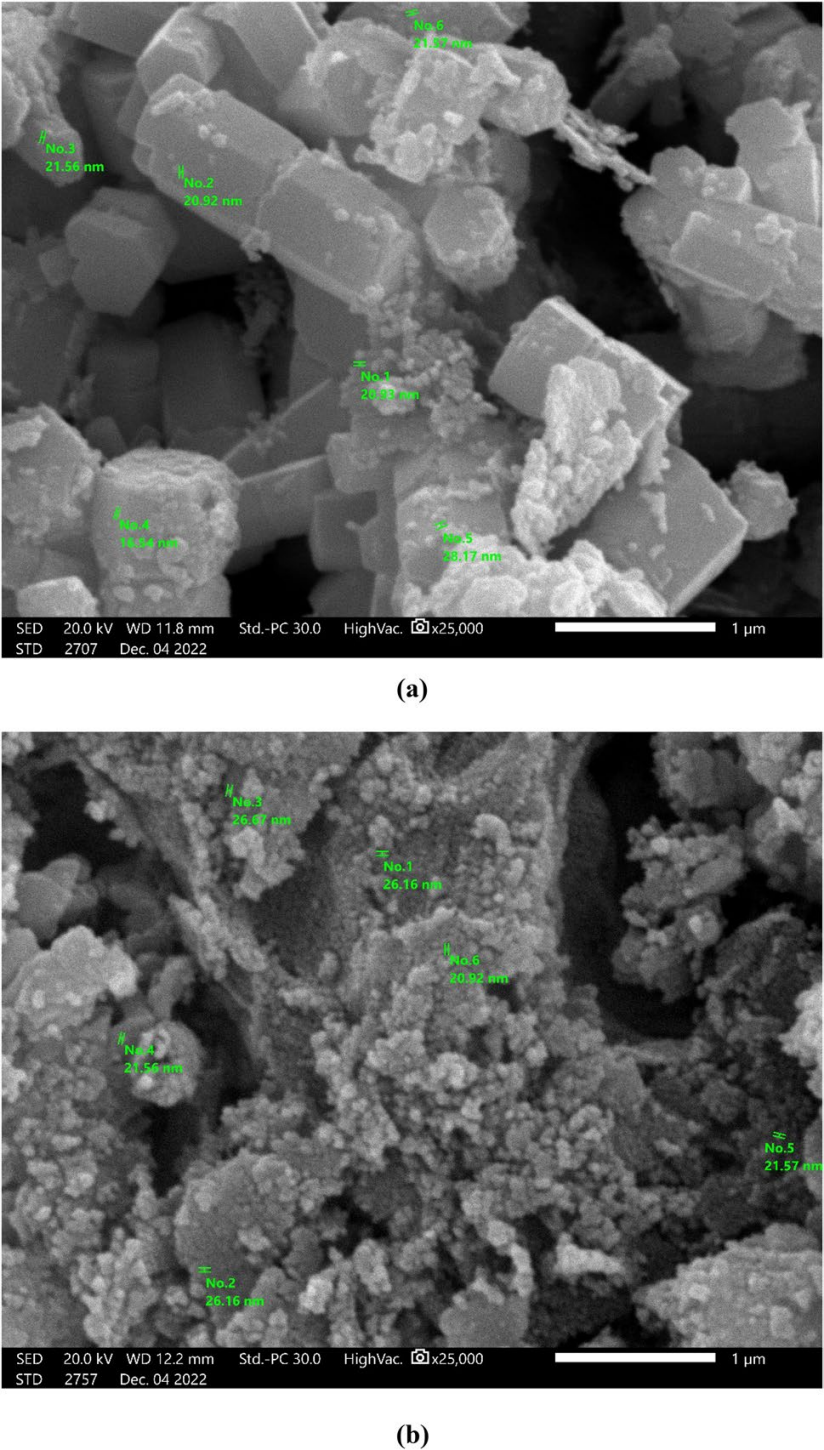
SEM image of (**a**) Green ZnO NPs, (**b**) Green 5% Co–ZnO NPs.

The field emission SEM device was applied at a magnifying power of 25,000 × to examine the surface morphology of ZnO NPs, which were synthesized using a green method. Images of synthesized ZnO NPs and Co–ZnO NPs are presented in Fig. 2a, b. The ZnO NPs shown in Fig. 2a as cube-shaped particles that appear to be aggregating together, however, this agglomeration was caused by the NPs accumulating on one another during SEM analysis. The Co–ZnO NPs surface morphology is depicted in Fig. 2b. The Co ions insertion into ZnO lattice positions may have an impact on how the morphology of the cuboid evolves to become bounded grains. Due to the doping of Co, the tiny particles have aggregated and become linked to an irregular shape. As shown in Fig. 2. SEM micrographs also reveal that Co–ZnO 5% NPs decrease in average particle size which can also be evident from our XRD measurements and TEM. This decrease in average particle size with increasing doping concentration was also reported by Godavarti et al.^31^.

### EDX analysis

**Figure 3:**
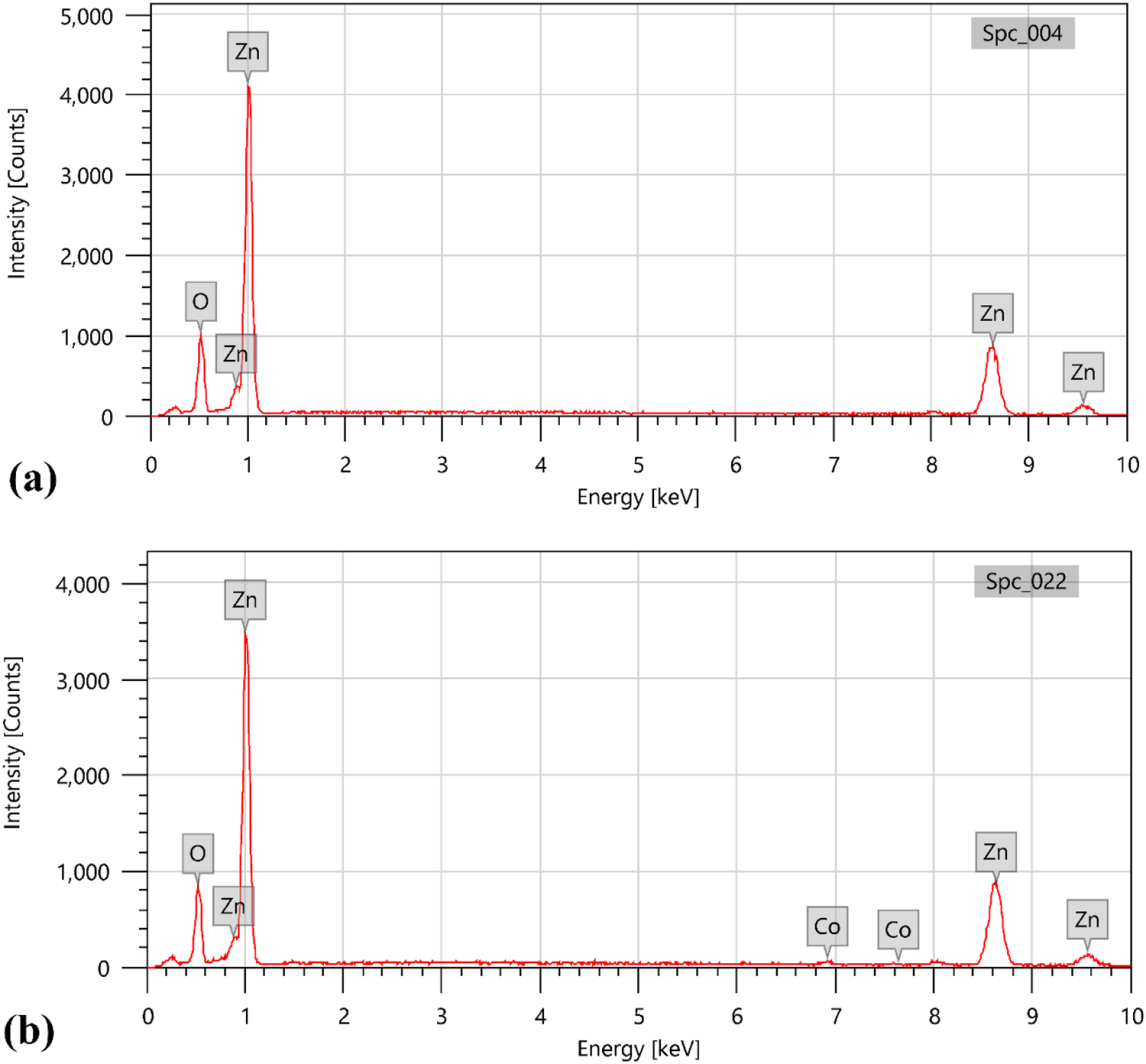
EDX bands of (**a**) Green ZnO NPs, (**b**) Green 5% Co–ZnO NPs.

Figure 3 displays the chemical compositions of produced Green ZnO NPs and 5% Co–ZnO NPs as found by EDX analysis. These spectra showed that just Zn, O, and Co were present (Table 3). The fact that the produced NPs include solely these components indicates that the Co^2+^ ions are replacing the Zn^2+^ ions in the Zn matrix, resulting in the peak of cobalt appeared in Fig. 3b. As seen in Fig. 3a, the only ions present in ZnO NPs are zinc and oxygen.

**Table 3:** Element analysis of Green ZnO NPs, and Green 5% Co–ZnO NPs using EDX analysis.

Element	ZnO NPs		5% Co–ZnO NPs	
	Mass%	Atom%	Mass%	Atom%
Zn	80.29 ± 0.90	49.93 ± 0.56	81.67 ± 0.92	53.34 ± 0.60
O	19.71 ± 0.24	50.07 ± 0.62	17.17 ± 0.23	45.83 ± 0.62
Co	0.0	0.0	1.15 ± 0.09	0.84 ± 0.06
total	100	100	100	100

### Transmission electron micrograph (TEM)

**Figure 4:**
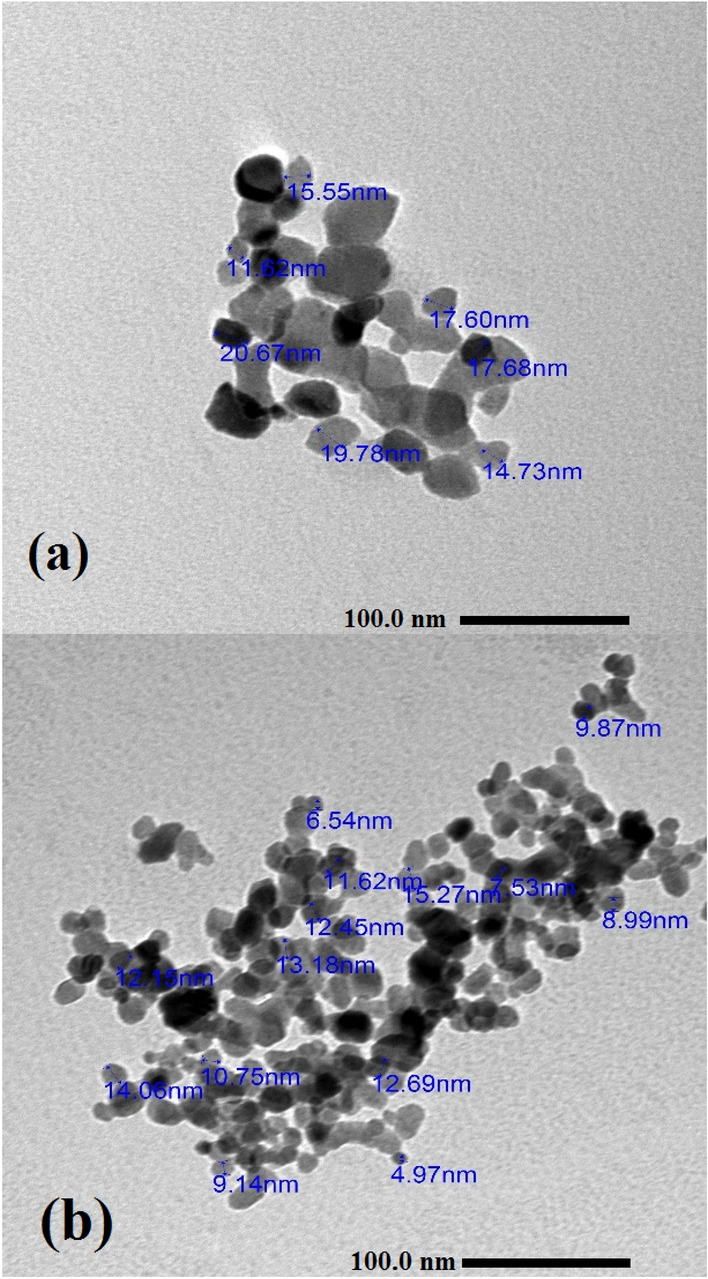
TEM images of (**a**) Green ZnO NPs, (**b**) Green 5% Co–ZnO NPs.

Figure 4 displays a TEM micrograph of zinc oxide NPs samples. These pictures show that the particles have a spherical and hexagonal form. There are some aggregated particles in the combination. Figure 4a, which depicts the TEM picture of ZnO NPs, shows that the size of the generated nanoparticles is less than 30 nm in diameter, which also supports the findings of the SEM examination. Most synthesized nanoparticles have spherical shapes, while some are slightly irregular. The synthesis of 5% Co–ZnO NPs with sizes ranging from 4 to 15 nm is confirmed by the TEM picture (Fig. 4b).

### X-ray diffraction (XRD) analysis

**Figure 5:**
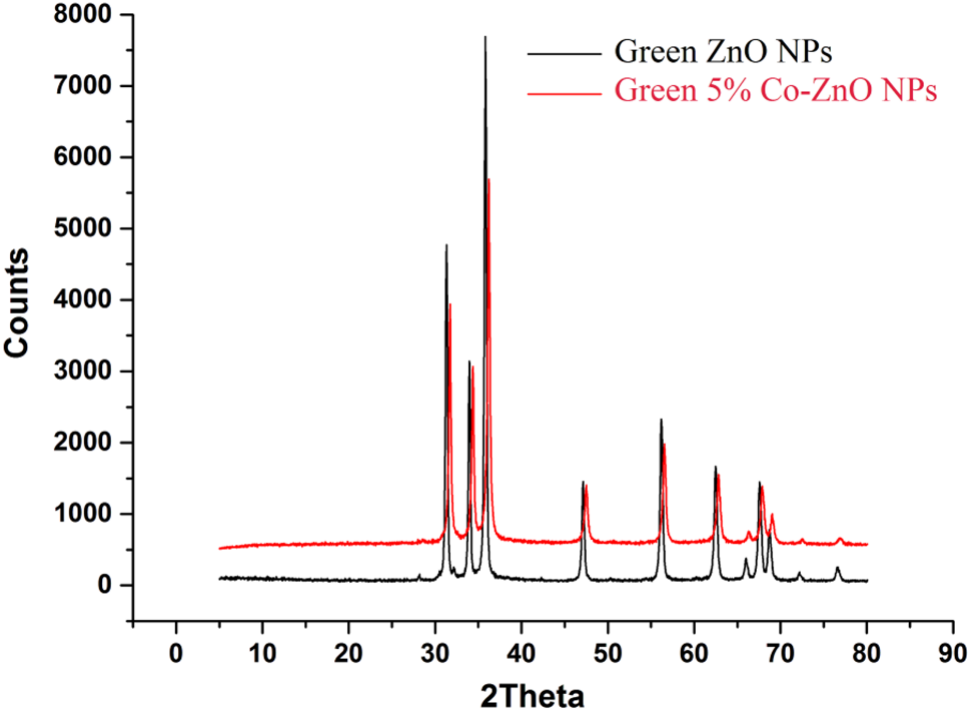
XRD of Green ZnO NPs and Green 5% Co–ZnO NPs.

Figures 5 and S2 show the XRD bands of synthetic Green ZnO NPs, 5, 10, and 15% Co–ZnO NPs, which were made using the aqueous extract of *P. capillacea* as a safe, environmentally friendly, and non-toxic solvent. A hexagonal wurtzite phase can be seen in the XRD bands of ZnO NPs and Co–ZnO NPs catalysts (Fig. 5). Moreover, cobalt-doped ZnO nanoparticles showed no secondary phase of cobalt or any other molecule, showing that the hexagonal ZnO phase was retained its present^22,53^. The diffraction bands of ZnO hardly changed after Co was doped into it. This is because, in response to a comparable ionic radius of cobalt (0.72) to that of zinc (0.74), cobalt ion replaces zinc without generating lattice deformation^41,54^. The very crystalline nature of the synthesized NPs is indicated by the strong diffraction peaks in Fig. S2. All the diffraction peaks have a hexagonal wurtzite structure as their index. Indicating a loss of crystallinity, the intensity of the XRD peaks dropped when cobalt content was raised. The Different relations used for the measurement of properties using XRD results were done according to the following calculation: Scherrer's formula was used^55^ for calculating the average crystallite size of nanoparticles (Eq. 3).

3 $$ {\text{D}} = \frac{0.9\lambda }{{\beta \cos \theta }} $$

where *D* is the average crystallite size, is X-ray wavelength, is full width at half maximum (FWHM) and is Bragg's angle.

Dislocation density ‘δ’ is the number of defect states in the sample and is calculated from crystallite size ‘D’ by the expression (Eq. 4):

4 $$\updelta =\frac{1}{{D}^{2}}$$

The microstrain (*ε*) can be calculated by using the following formula (Eq. 5).

5 $$\varepsilon =\frac{ \beta \mathrm{cos}\theta }{4}$$

*β* is Full width at half maximum (FWHM) and is Bragg's angle.

The lattice parameters for all hexagonal wurtzite structured samples are calculated by using the following Eq. (6).

6 $$\frac{1}{d2}=\frac{4}{3} \left(\frac{{h}^{2}+hk+{k}^{2}}{{a}^{2}}\right)+\frac{{l}^{2}}{{c}^{2}}$$

where *d* is interplanar spacing, *h*, *k* and *l* are Miller indices, and *a* and *c* are lattice parameters. Considering the first order approximation (*n* = 1) for the (100) plane, the lattice constant ‘a’ is obtained through the relation (1), and Lattice constant ‘c’ is derived for the plane (004) by the relation (2) and The volume of the unit cell was calculated by using following Eqs. (7–9):

7 $$\mathrm{a}=\frac{\lambda }{\sqrt{3 }\mathrm{sin}\theta }$$

8 $$c=\frac{\lambda }{\mathrm{sin}\theta }$$

9 $$V=\frac{\sqrt{3 } {a}^{2}c}{2}$$

The Scherrer-Debye Eq. (3) and the previously specified data are used to determine the crystal size. Where, k = Shape factor (0.94), = Full width at half maxima of peak (FWHM), = X-Ray wavelength, and = Diffraction Angle (radians). For doping levels of 0, 5, 10, and 15% Co, the measured crystallite sizes are found to be 46.02, 45.81, 42.82, and 43.95 nm, respectively (Table S2). It depicts the considerable alterations in the diameters of the pure crystallites of ZnO caused by doping. The crystallite size reduced from 46.02 to 43.95 nm when the Co doping concentration increased from 0 to 15%. The outcome indicates that the average crystallite size has reduced as co-doping levels have increased. The distortion in the host is the main cause of the decrease in crystallite size. The distortion of the host lattice is caused by foreign contaminants (i.e., Co^2+^), which inhibits the nucleation and subsequent growth of ZnO NPs, this is the main cause of the decrease in crystallite size^55^. Additionally, the XRD peak intensity decreased with increasing Co concentration, a symptom of diminishing crystallinity. This may be because of the substitutional absorption of Co ions into the lattice of ZnO NPs, which may cause a reduction in the orientation of the ZnO host lattice. The lattice imperfections brought on by the different ionic radii of Co and Zn can also lessen the intensity of the peak. It is possible to infer from the shift in peak intensities that the doping is successful and that Co is integrated into the lattice^56^. The variation of lattice parameters a and c with Co molar concentration is shown in Table S3. There is a very small change observed in lattice parameters of ZnO with increasing Co content. This result is attributed to the systematic substitution of Zn^2+^ by Co^2+^ ions without disturbing the crystal structure of ZnO^36^. This may also be due to nearly the same ionic radii of Zn^2+^ and Co^2+^ ions. The investigation of change in the lattice strain (ε) that arises due to the addition of Co to ZnO. Usually, the increment/decrement in lattice strain with dopant concentration is related to the increment decrement in the dislocation density^55^. In the present work also the increase in the lattice strain on doping is associated with the increase in dislocation density (Table S3). A small shift is also observed in the peaks for the cobalt-doped ZnO samples due to the replacement of cobalt Co^2+^ with Zn^2+^ evident as a decrease of intensity peak (101) as shown in Figs. 5 and S2. Evidence of peak reduction in terms of peak intensity can influence particle size or lattice strain. Table S3 shows the variation in average crystallite size and strain for cobalt concentration for Co–ZnO 5% NPs. The changes produced in strain are sufficient to produce the formation of clusters or precipitation which could be further evident using TEM and SEM. Deformation variation is observed based on an increase in lattice parameters (a, c) and volume as evident in Table S3. The smaller variation observed is attributed to a mismatch in the radius of cobalt and zinc oxide. The lattice parameters are expected to increase due to cobalt doing as assuming tetrahedral environment with high spin state for Co^2+^ (0.745 Å) and low spin state for Co^2+^ (0.65 Å) are smaller than Zn^2+^ (0.60Å) as reported elsewhere^55^. The increase in volume (Table S3) resulted in a change in oxygen parameter, which increases with an increase in cobalt. These increases in defect density can cause a change in the lattice parameters of ZnO.

### UV–Vis and DRS analysis

**Figure 6:**
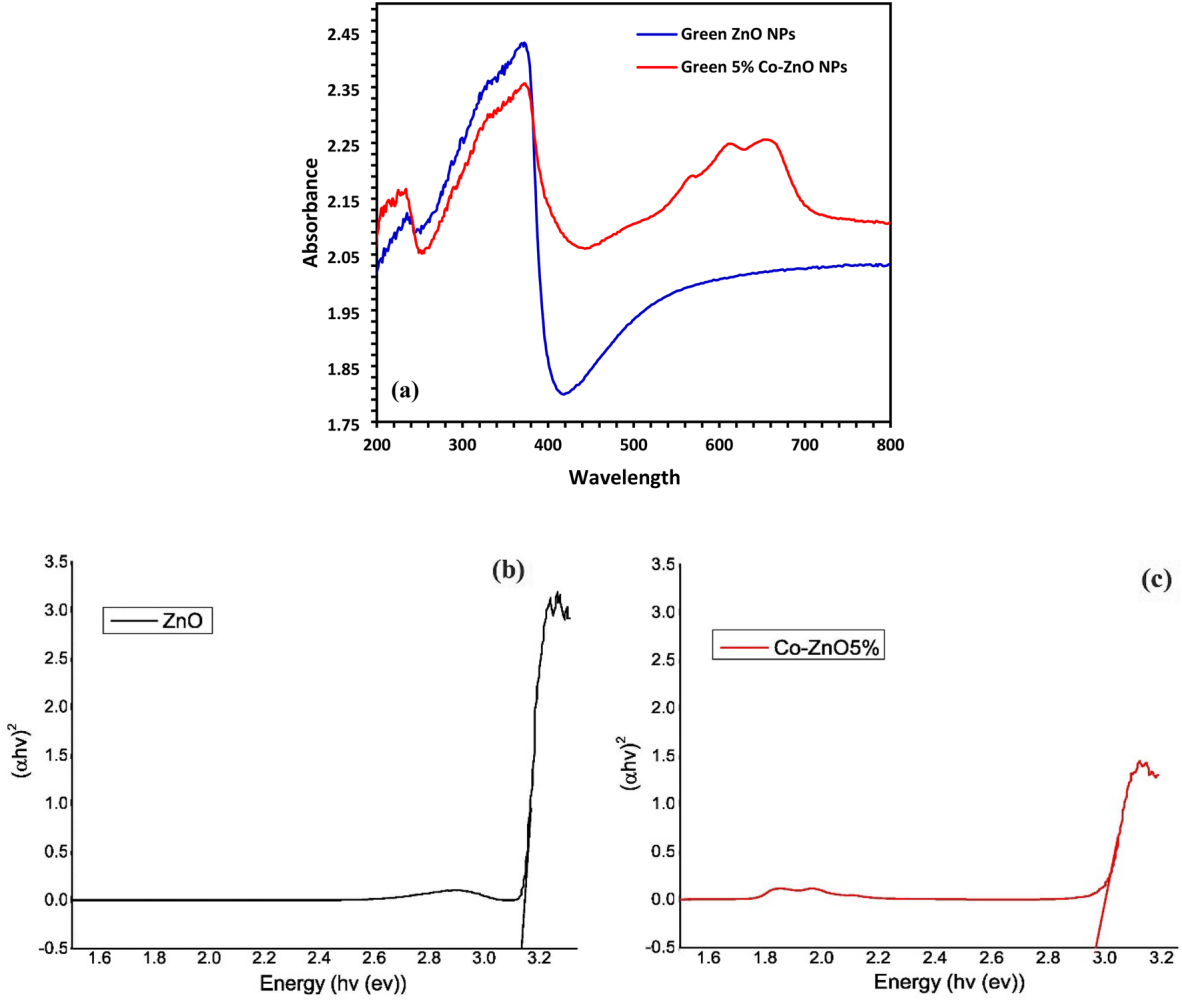
(**a**) UV–DRS spectra of Green ZnO NPs and Green 5% Co–ZnO NPs, (**b**) Tauc plots of Green ZnO NPs and (**c**) Green 5% Co–ZnO NPs.

The UV–Vis spectra were used to investigate the Green 5% Co–ZnO NPs and Green ZnO NPs catalysts' optical properties. The spectra of the 5% Co–ZnO NPs and ZnO NPs catalysts are shown in Fig. 6a. By making a comparison to pure ZnO, which only absorbs in the UV region (λ < 400 nm), which corresponds to the band gap transition of ZnO structure. The Co–ZnO NPs exhibit substantial visible-range absorption (λ > 400 nm), which causes the band gap energy value to drop. Three absorption bands are shown in the transmission spectra 570 nm (2.175 eV), 612 nm (2.026 eV), and 655 nm (1.893 eV) which are related to d–d transitions of Co^2+^ ions. These absorptions result from d–d transitions of high spin Co^2+^ ions^22^, which could be assigned to transition from ^4^A2 → ^2^E (G), ^4^A2 → ^4^T1(P), and ^4^A2 → ^2^A1(G), respectively^22^. This result indicates the successful substitution of Co^2+^ into Zn^2+^ sites where no CoO or Co_3_O_4_ phase has been indicated from the XRD results. The reason for this reduction may be due to oxygen defects, which may be the result of sp–d exchange interactions between band electrons and localized d electrons of Co^2+^ ions. These interactions could lead to the introduction of new energy levels as well as an excess of free electrons across the valence band. As a result, this discovery raises photocatalysis activity under visible light^22^. Regarding the Co-doped ZnO NP sample. The absolute intensity of these absorption bands increased approximately linearly with increasing cobalt concentration. Since the ionic radius of Co^2+^ in the tetrahedrally coordinated structure is like that of Zn^2+^ (74 nm), this behavior can be recognized by the d–d transitions of the molecule. These transitions cause the sample's color to change from white to green^57^. The energy bandgap (Eg) was calculated using Tauc’s relation as follows (Eq. 10)^58^.

10 $$ \alpha h\vartheta = A\left( {h\vartheta - Eg} \right)^{n} $$

Here, ‘*A*’ is a constant, ‘*α*’ is the absorption coefficient, and ‘*n*’ is a constant equal to 2 for an indirect transition and 1/2 for a direct transition. The (*αhυ*)^2^ values against photon energy (*hυ*) are represented in Fig. 6b and c. An optical direct energy bandgap (E_g_) values were found to be 3.19 and 2.92 eV for the Zn1-xCoxO NPs with x = 0.00, and 5% of Co, respectively. It was observed that the Eg value reduced with the rise in Co concentration, as suggested by the redshift in absorption spectra. A comparable reduction in the bandgap energy with Co level has been reported by^58,59^.

### X-ray photoelectron spectroscopy (XPS)

**Figure 7:**
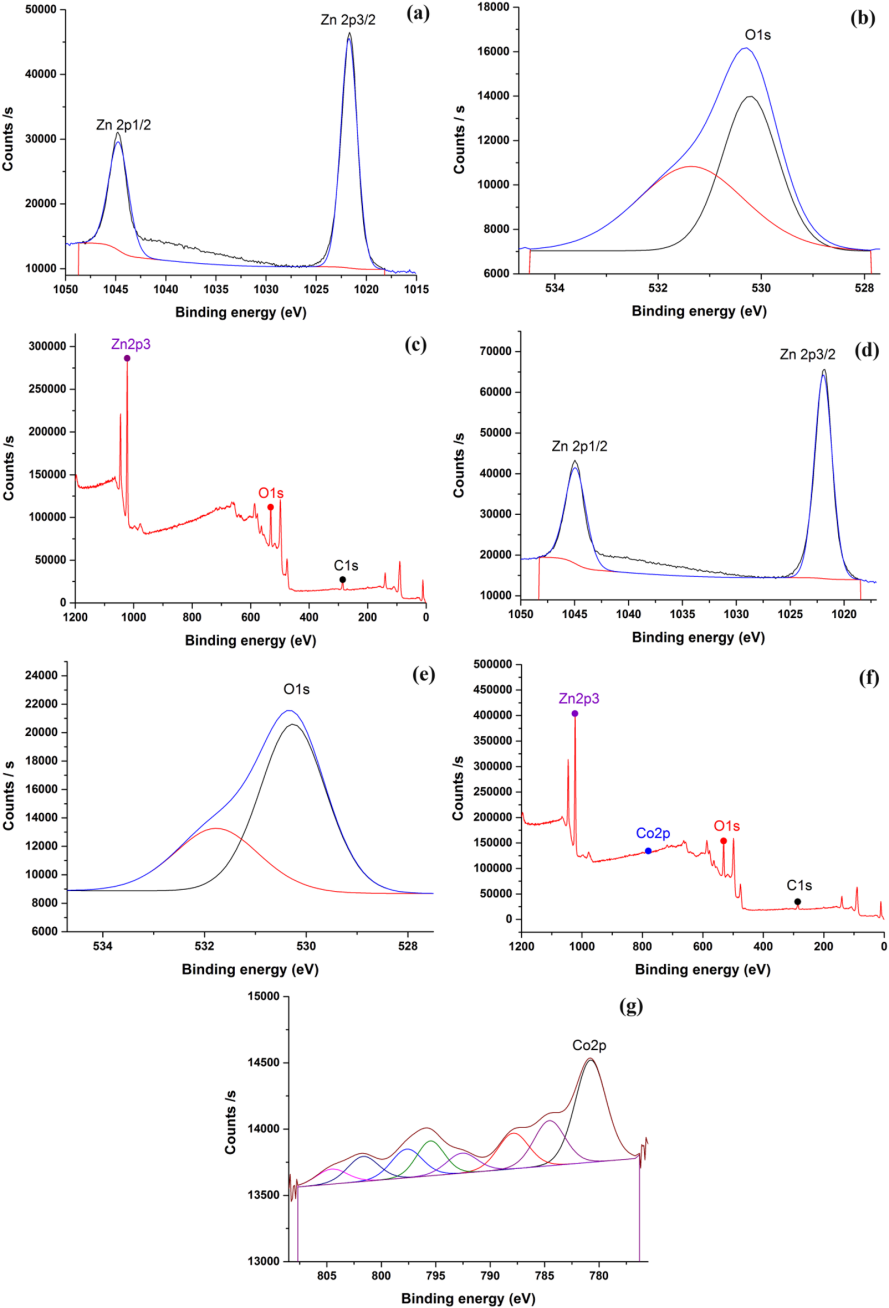
X-ray photoelectron spectra of (**a**–**c**) Green ZnO NPs, (**d**–**g**) Green 5% Co–ZnO NPs.

To investigate the Zn and Co content and their valence states, XPS measurements were used (Fig. 7). Where standard C1s spectra showed that reference binding energy of 285.84 eV. For the ZnO NPs, the Zn core level breaks into Zn 2p_3/2_ and Zn 2p_1/2_ at 1021.72 and 1044.71 eV, respectively (Fig. 7a, b, c). The measurements match those of the normal ZnO sample, and no noticeable peak shift is seen. This demonstrates that Zn is present in the ZnO lattice in the 2 + oxidation state^60^. According to Fig. 7a's O1s spectrum, the peak at 530.22 eV is associated with O^2−^ ions surrounded by Zn^2+^ ions in the ZnO structure, while the peak at 531.7 eV is caused by chemisorbed or dissociated oxygen or [OH^–^] species on the surface (OC)^60^. Figure 7d, e, f, g shows the XPS analysis of the 5% Co–ZnO NPs sample. There are peaks of Co 2p_3/2_ at 780.77 eV, and Co 2p_1/2_ at 794.21 eV with satellite peaks (Fig. 7g). The divalent state of cobalt, which is homogeneously bound by oxygen atoms in tetrahedral coordination, is shown by a 13.44 eV energy difference between Co 2p_3/2_ and Co 2p_1/2_.

### Thermal analysis (TGA)

**Figure 8:**
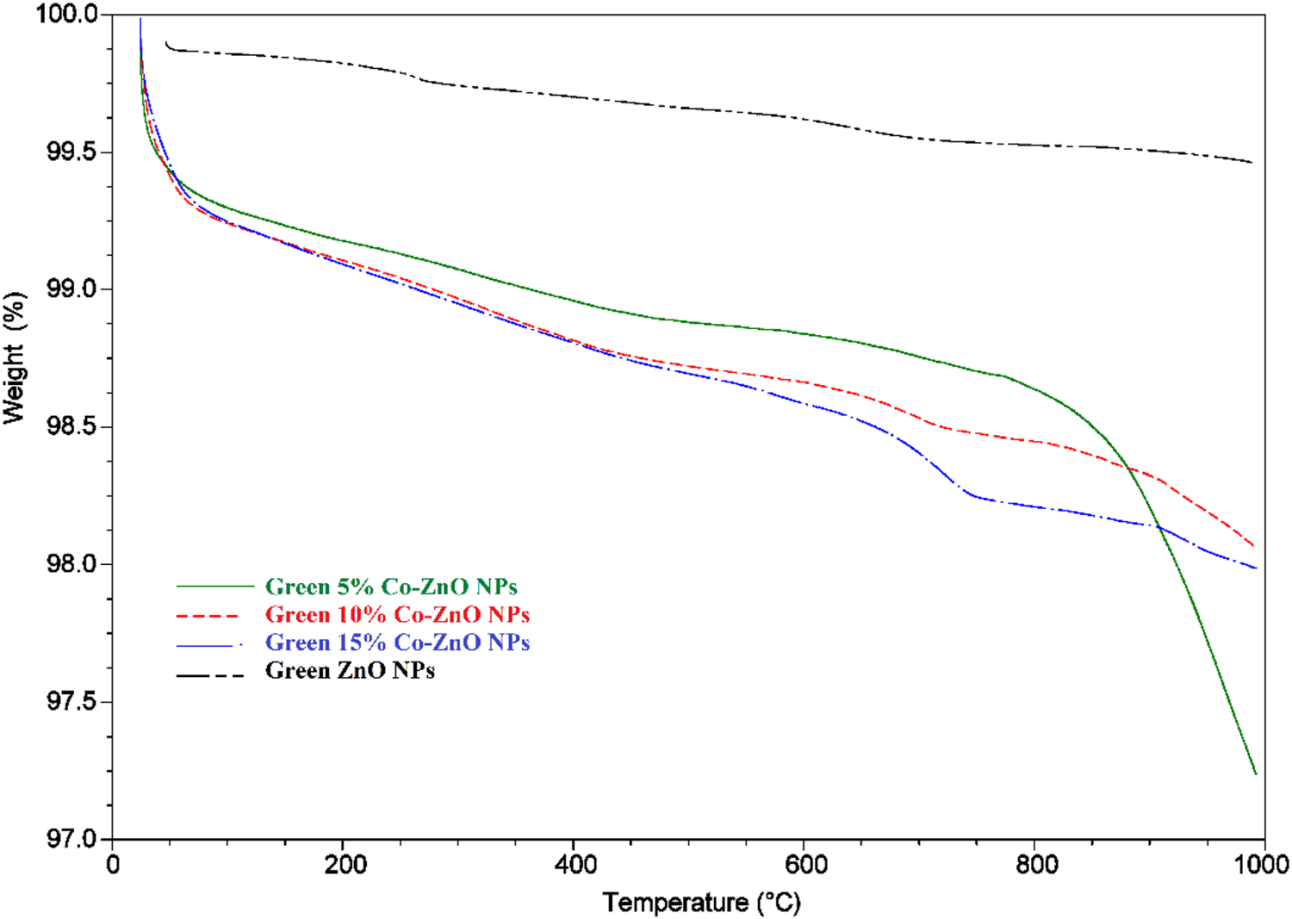
TGA analysis of Green ZnO NPs, Green 5, 10, and 15% Co–ZnO NPs at temperatures ranging from 50 to 1000 °C.

The physical properties and constituents of the Green 5% Co–ZnO NPs and Green ZnO NPs are revealed by their thermal stability (Fig. 8). Figure S3 displays the weight loss for ZnO NPs and cobalt-doped ZnO NPs samples with the TGA spectra up to 1000 °C. The findings show that whereas the ZnO sample was essentially unaltered, the 5% Co–ZnO NPs sample lost weight abruptly. An initial weight loss was seen for the 5% Co–ZnO NPs and ZnO NPs samples between 50 and 250°C because of the sample's loss of moisture and organic materials. The leftover precursors continued to be eliminated and emitted as CO_2_ between 250 and 900 °C^51^. Around 900 °C, the weight loss seemed to stabilize, indicating that the majority of pollutants had been eliminated by this time. The weight loss in the ZnO samples was minimal and was mostly caused by moisture degradation and CO_2_ absorption.

## Photocatalytic activity

### Photocatalytic test

**Figure 9:**
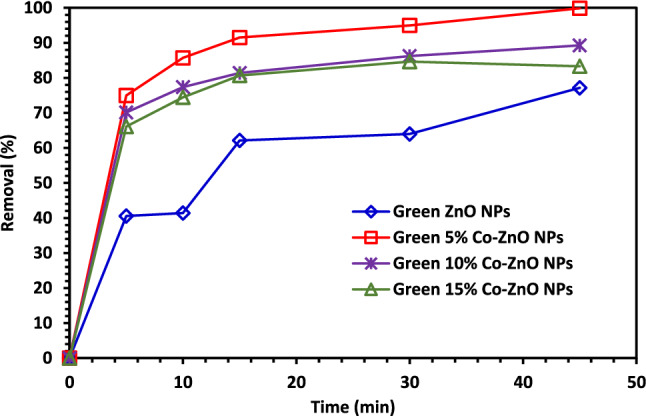
Photocatalytic test of CIPF with Green ZnO NPs, 5, 10, and 15% Co–ZnO NPs.

According to the best performance, it was found that 5% Co–ZnO NPs gave the best performance with the removal of 99% after 45 min of CIPF as shown in Fig. 9.

#### Determination of point of zero charge (pH_PZC_) of 5% Co–ZnO NPs

**Figure 10:**
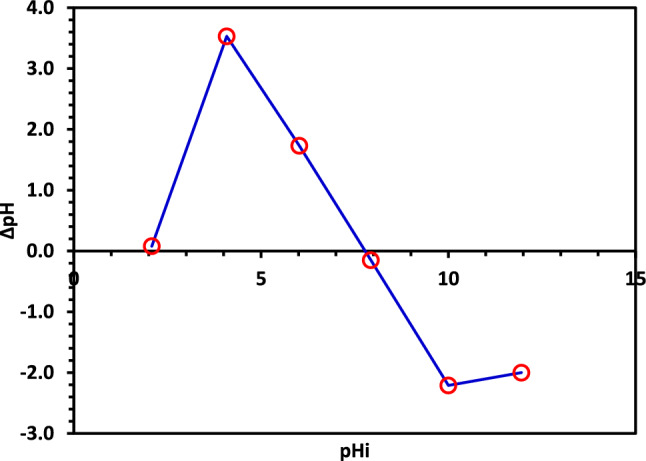
The pH_PZC_ determination of the Green 5% Co–ZnO NPs.

The pH_PZC_ is a fundamental indicator of material properties and is measured to determine the net charge of a catalyst as a function of the pH of a solution. Furthermore, determining pH_PZC_ is a crucial first step in examining how the photodegradation process of the catalyst changes as the pollutant solution changes pH. In the current work, six flasks containing a precise quantity of this catalyst (100 mg) and 50 mL of 0.1 N NaCl solution were used to test the pH_PZC_ of 5% Co–ZnO NPs. By adding HCl and NaOH (0.1 N) dropwise, the beginning pH values of these solutions were changed at 2, 4, 6, 8, 10, and 12. The flasks were then shaken for 24 h at 150 rpm. The final pH of each solution was determined following the shaking procedure. The pH_PZC_ value of 5% Co–ZnO NPs is represented by the intersection of the beginning pH curve and the pH (the difference between the initial and final pH) curve (Fig. 10).

#### The effect of pH on CIPF photodegradation.

**Figure 11:**
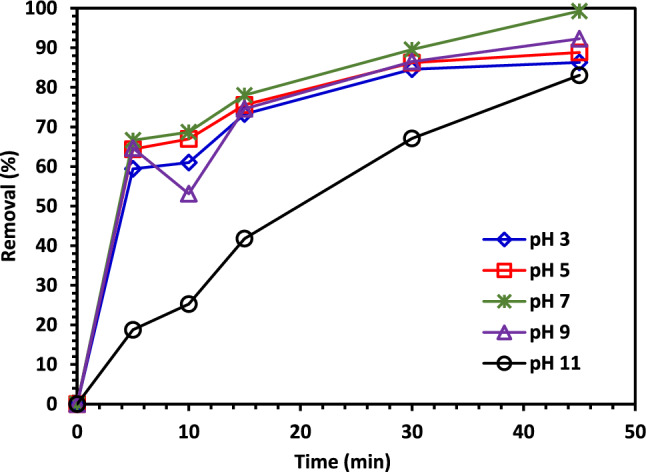
The impact of beginning pH on CIPF photodegradation using Green 5% Co–ZnO NPs as a catalyst.

The solution pH was varied to 3, 5, 7, 9, and 11, the initial concentration of CIPF (30 ppm), and the catalyst dose (100 mg) to study the impact of the initial pH of the solution on the CIPF photocatalytic degradation. The results in Fig. 11 show that the pH of nature (pH = 7) is the optimal pH for the decomposition of CIPF. The effect of initial pH on the photodegradation of pollutants is complex, with results generally dependent on the type of pollutant and the zero-point charge (pH_PZC_) of the photocatalyst. The pH of the solution affects the photocatalyst's surface charge characteristics; thus it has a significant impact on the electrostatic interaction between the catalyst surface and the contaminant particles. The effect of pH on the CIPF antibiotic degradation can be examined by looking at the properties of both the catalyst and the antibiotic at various pH values. For Co–ZnO NPs, the pH_PZC_ is 7.8 (Fig. 10), thus, the catalyst surface is positively charged at pH < 7.8 and negatively charged at pH > 7.8^61^. On the other hand, CIPF has a pka of 6.09 and 8.2. The adsorption on the surface of Co–ZnO NPs is constrained at acidic pH because both Co–ZnO NPs and CIPF are positively charged at pH values more than 6.09. The Co–ZnO NPs have a positive surface, while the surface of CIPF is negative, leading to antibiotic adsorption on the surface of Co–ZnO NPs and an increase in the rate of degradation. When a solution's pH is more than 7.8, CIPF will take on an anionic form (CIPF-O^–^), which prevents species from oxidizing and ultimately reduces the effectiveness of CIPF removal^61^.

#### The impact of Catalyst dosage on CIPF photodegradation

**Figure 12:**
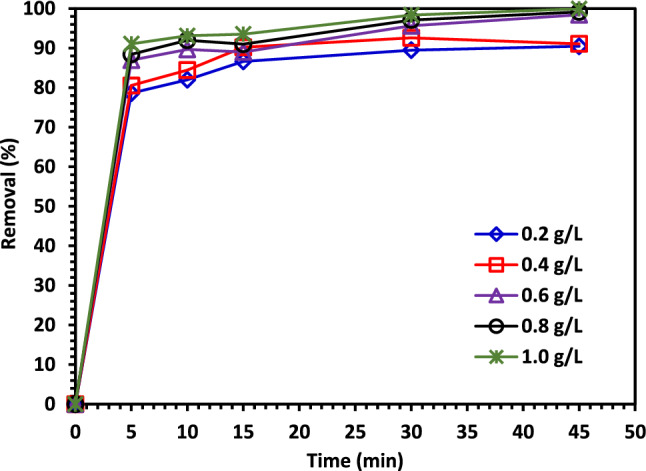
The impact of Green 5% Co–ZnO NPs dosage on the photodegradation of CIPF.

By changing the catalyst loading from 0.02 to 0.10 g/100 mL while keeping all other variables constant, the effect of catalyst (5% Co–ZnO NPs) doses on photocatalytic degradation of CIPF under visible light illumination was systematically examined. Figure 12 demonstrates how the amount of CIPF that is degraded increases as the catalyst dose is increased; however, as the catalyst dosage is increased further, the amount of antibiotic that was degraded decreases. The growth in the number of active sites for the adsorption of CIPF molecules on the catalyst surface as well as the capacity of light absorption to produce an increasing number of radicals at catalyst surfaces could be responsible for the increase in photocatalytic activity with increased catalyst dosage^62^. The decrease in photocatalytic activity that happens with higher catalyst doses may be caused by the aggregation of nanoparticles, which blocks light from penetrating as well as adsorption by accumulated catalysts^62,63^. It was discovered that 1.0 g/L was the ideal dose level for the increased photocatalytic activity.

#### The impact of CIPF initial concentration

**Figure 13:**
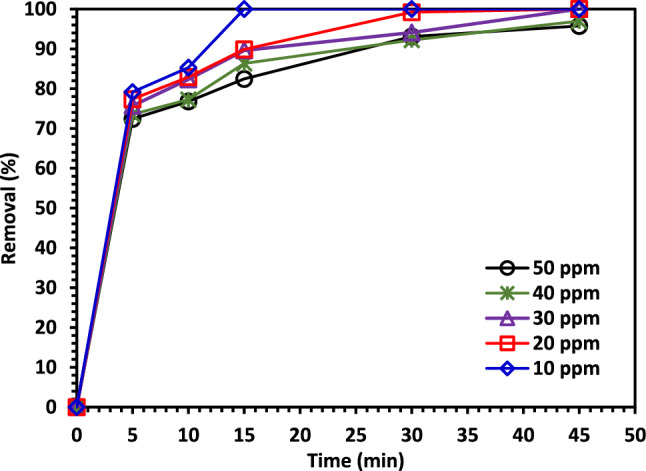
The impact of initial CIPF antibiotic concentration on its photodegradation using Green 5% Co–ZnO NPs.

The CIPF degradation at various initial concentrations (10, 20, 30, 40, and 50 ppm) was examined and is depicted in Fig. 13 for a catalyst dose of 1.0 g/L of 5% Co–ZnO NPs catalyst. The efficiency was inversely proportional to the increase in CIPF concentration. This is because the equilibrium adsorption of the antibiotic on the active catalyst surface sites increases with antibiotic concentration, resulting in a reduced rate of OH radical production, which is the process' primary oxidant^64,65^. The greatest degradation of CIPF was determined to be 100% at 10 ppm and 82% at 50 ppm. This shows that CIPF needs more time to degrade at higher concentrations.

#### The temperature impact on the CIPF degradation

**Figure 14:**
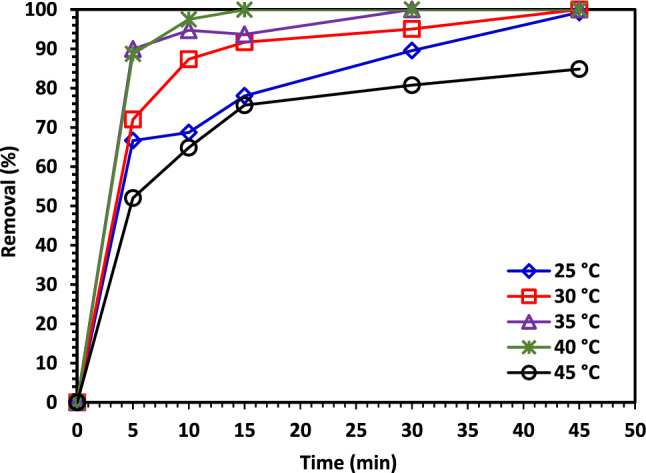
The temperature impact on the photodegradation of CIPF using Green 5% Co–ZnO NPs.

As the temperature has been shown to improve catalyst performance, it was anticipated that the removal of CIPF by 5% Co–ZnO NPs would rise as the temperature was raised. The elimination of CIPF by 5% Co–ZnO NPs was examined in the current study at various temperatures, including 25, 30, 35, 40, and 45 °C (Fig. 14). The increased rate of $^{ \cdot } {\text{OH}}$ production appeared to be the cause of the 5% Co–ZnO NPs process' improved removal effectiveness of CIPF at increased temperature. A further explanation that was considered was that at higher temperatures, more target molecules would gain activation energy and breach the energy barrier, which would then cause them to collide with reactive radicals^66^. The removal efficiency in this study increased from 25 to 40 °C; when the temperature rises, the catalyst's activation rises, leading to higher CIPF degradation efficiency. Degradation efficiency is decreased at higher temperatures (45 °C) because the radicals interact with one another rather than the ciprofloxacin molecule^67^.

#### Effect of shaking speed

**Figure 15:**
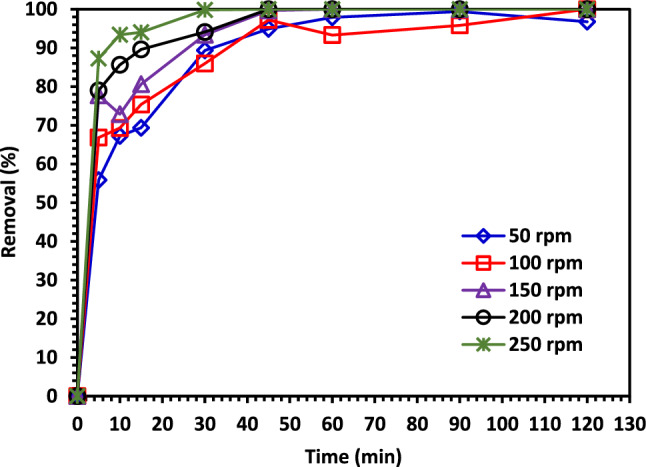
The shaking speed impacts the photodegradation of CIPF using Green 5% Co–ZnO NPs.

The rate at which pollutant molecules come into contact with photocatalyst particles depends directly on the degree of mixing. Figure 15 depicts the impact of a 50 to 250 rpm shaking speed increase on the CIPF under the following conditions: pH = 7, 5% Co–ZnO , 1.0 g/L, CIPF initially concentration, 30 mg/L, 50–250 rpm shaking, and 25 °C are all acceptable values. Because the mixing rate of catalyst particles in the aqueous solution rose with an increase in shaking speed, the CIPF photodegradation was improved. The speed at which the pollutant molecules collided with the catalyst particles and interacted with them increased as a result of the enhanced photodegradation rate. Furthermore, when the rate of aqueous solution-to-catalyst particle mixing increased, the boundary layer on the solid particles shrank, reducing the resistance of the thin layer surrounding the catalyst particles to mass transfer^68^.

#### The scavengers' impact on removal efficiency

**Figure 16:**
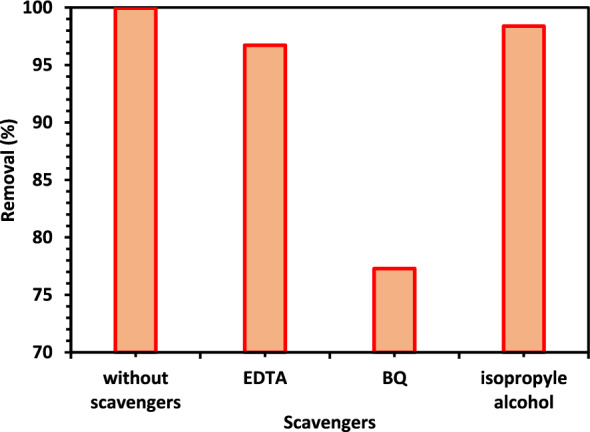
Different scavengers impact on photodegradation of CIPF in the existence of Green 5% Co–ZnO NPs (1.0 g/L), reaction pH = 7.

The goal of the scavenging investigation is to identify the primary photo-catalytically active species so that the mechanism of CIPF photocatalytic degradation by 5% Co–ZnO NPs can be assessed. Utilizing 5% Co–ZnO NPs, CIPF is photo-catalytically degraded utilizing an advanced oxidation method. The procedure primarily entails the creation of a (e^−^)-hole photoelectron pair. In subsequent phases, this (e^−^)-hole pair produces extremely reactive O_2_^–·^ and ^·^OH radicals once more, leading to the oxidative destruction of the antibiotic^64,65^. Additionally, hydrogen atoms produced by water contribute to the antibiotic's reductive decomposition. By adding a scavenger to the CIPF solution during photocatalysis, the scavenging study was carried out in identical conditions. Benzoquinone (BQ) (1.0 mM), isopropanol (IPA) (10 mM), and Na-EDTA (10 mM) are used to capture oxidative species such as ^·^OH, h^+^, and O_2_^–·^. These are employed, in turn, as OH scavengers, O2-scavengers, h^+^ scavengers, and OH and h^+^ scavengers. Results for CIPF degradation by 5% Co–ZnO NPs with and without scavengers are shown in Fig. 16. The percentage of CIPF that is photo catalytically degraded by the addition of Na-EDTA, IPA, and BQ drops to 96.7, 98.3, and 77.3%, respectively.

#### Kinetics of CIPF photodegradation

**Figure 17:**
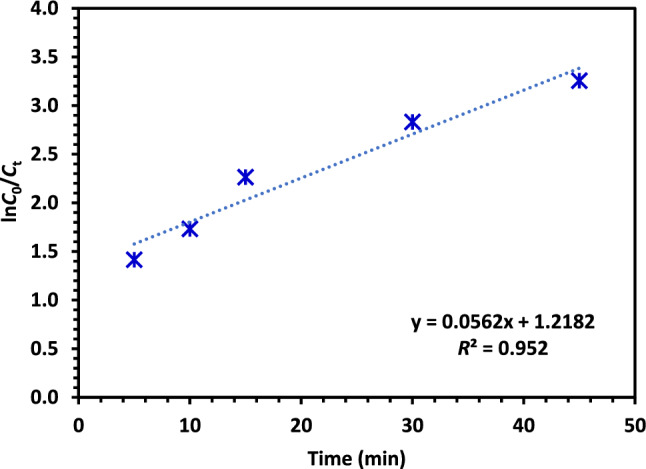
Kinetics of CIPF photocatalytic degradation using Green 5% Co–ZnO NPs.

The reaction kinetics of the CIPF photocatalytic degradation were tested using the Langmuir–Hinshelwood (LH) model. Many scientists used the first-order expression as an approximation of the model to describe how antibiotic contaminants are removed using visible light. The physical adsorption of the pollutant on the surfaces of the photocatalysts, a surface reaction that develops into antibiotic degradation in the presence of visible light, and the formation of additional by-products are just a few of the complex steps that reactions involving heterogeneous catalysts can involve^69,70^. Figure 17 shows the diagram of the LH model. As shown in Eq. (4), the value of the model's rate constant (k) may be determined directly from the slope of the plot.

11 $$\mathrm{ln}\frac{{C}_{0}}{{C}_{t}}=kt$$

where *C*_0_ is the initial concentration of CIPF, *C*_t_ is the concentration of CIPF at time *t* taken for photodegradation in minutes. According to the LH model plot and value produced, the removal of CIPF antibiotics best matches the first order of the LH model precisely, with a linear correlation of 0.952. The pathway of photocatalytic degradation is predominant in the removal of CIPF antibiotics (*k*_obs_ = 0.0562 min^−1^).

#### Photocatalytic stability

**Figure 18:**
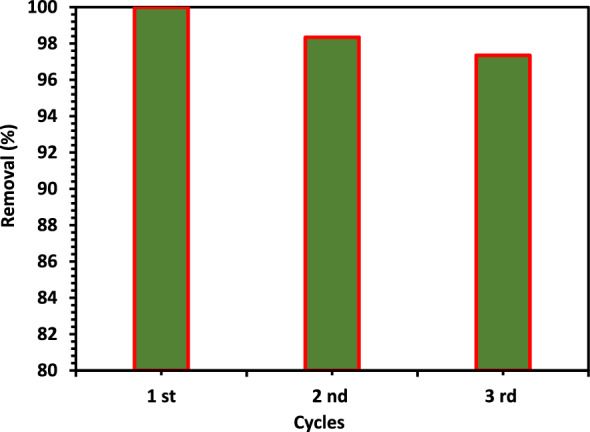
Recyclability of Green 5% Co–ZnO NPs within three successive cycles for the CIPF photodegradation under irradiation with visible light.

Figure 18 shows the stability investigation of the 5% Co–ZnO NPs sample. The same 5% Co–ZnO NPs photocatalyst was used three times to examine the stability of the catalyst under identical experimental circumstances. The degrading efficiency of CIPF has not changed much after three consecutive cycles. The degradation percentage vs. cycle number plot is presented in Fig. 18, and it can be seen from the plot that the degradation percentage of the CIPF antibiotic has slightly changed. This decrease in degradation percentage, however, may be brought on by the 5% Co–ZnO NPs photocatalyst producing a hydroxide layer on its surface, or it may be linked to the loss caused by the catalyst's inevitable leaching during the recovery and washing process, as well as the blocking of the active site with CIPF antibiotic and its degradation intermediates on the surface^71^. The 5% Co–ZnO NPs photocatalysts stability and recyclability were both demonstrated to be good.

#### Mechanism of photodegradation of CIPF

**Figure 19:**
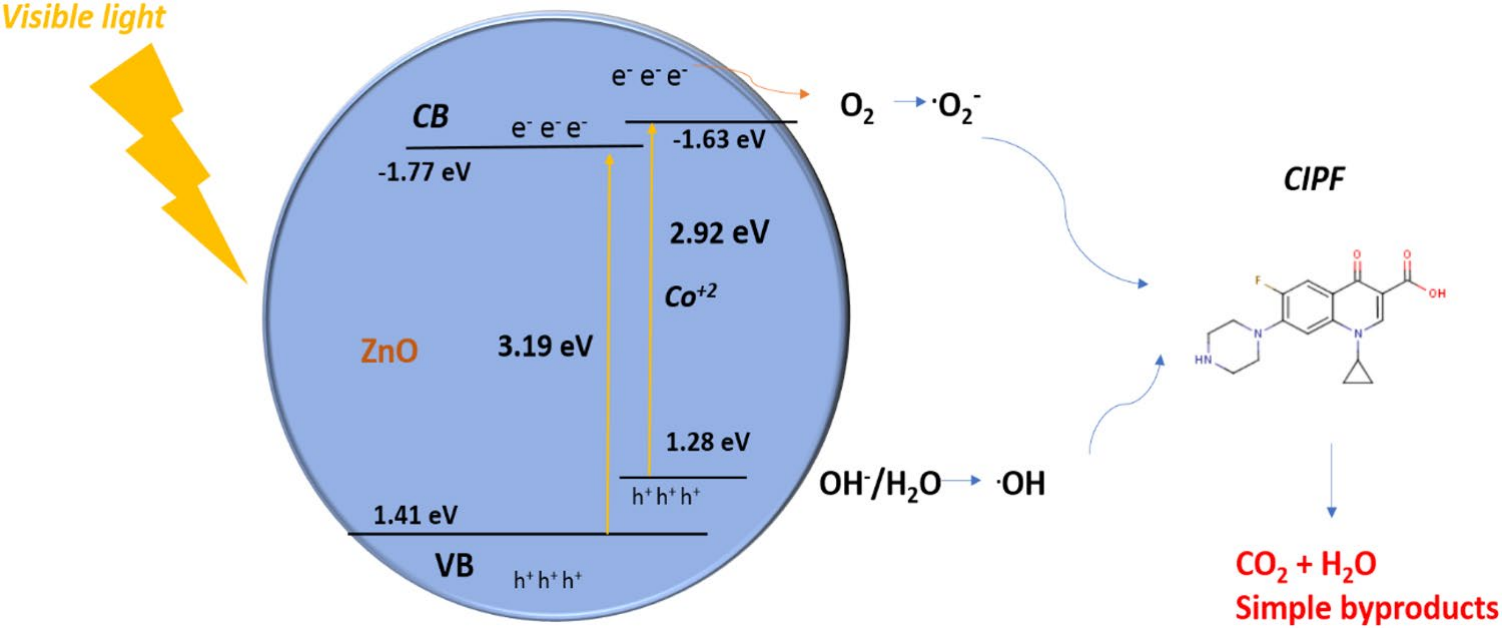
The mechanism of photocatalytic degradation using Green 5% Co–ZnO NPs catalyst.

The potential photodegradation mechanism of ciprofloxacin is also specified in Fig. 19. Due to the photo-generated separation of electrons and holes and the expansion of the light absorption spectrum, the photocatalytic activities of 5% Co–ZnO NPs were improved ^41^. After being exposed to visible light, the photogenerated electrons may go to the Co–ZnO conduction band. While the remaining holes in Co–ZnO 's valence band are predicted to undergo a reaction with OH^−^ and H_2_O to create reactive oxygen species (^·^OH), photogenerated electrons in the conduction band of the material are predicted to be trapped by oxygen and converted to superoxide radical anions (O_2_^−^). The main species involved in hydrolyzing CIPF are ^·^OH and ^·^O_2_^−^^41,72^. The generation of superoxide free radicals (^**.**^O_2_^–^) was aided by electrons (e) in the conduction band. They were combined to form the H_2_O_2_ molecule due to the highly reactive nature of reactive oxygen species (ROS). The ciprofloxacin was degraded as the H_2_O_2_ molecule was disintegrated into ^**.**^OH free radicals. The chemical equations for ciprofloxacin degradation through active species are given below:

12 $$ {\text{Co}} - {\text{ZnO}} + h\upsilon \to {\text{h}}^{ + } \left( {{\text{VB}}} \right) + {\text{e}}^{-} \left( {{\text{CB}}} \right) $$

13 $${\text{h}}^{ + } \left( {{\text{VB}}} \right) + {\text{H}}_{{2}} {\text{O}} \to {\text{H}}^{ + } + ^{ \cdot }{\text{OH}}$$

14 $${\text{e}}^{ - } \left( {{\text{CB}}} \right) + {\text{O}}_{{2}} \to {\text{CB }} + ^{.}{\text{O}}_{{2}}^{-}$$

15 $$^{.} {\text{O}}_{{2}}^{-} + {\text{ H}}^{ + } + {2}^{.} {\text{OH}} \to {\text{O}}_{{2}} + {\text{ + 2 H}}_{{2}} {\text{O}}_{{2}} $$

16 $$ {\text{2H}}_{{2}} {\text{O}}_{{2}} \to {4}^{.} {\text{OH}} $$

17 $$^{.} {\text{O}}_{{2}}^{-} + ^{.}{\text{OH}} + {\text{CIPF}} \to {\text{CO}}_{{2}} + {\text{H}}_{{2}} {\text{O}}$$

#### Optimization of Co–ZnO NPs process conditions by RSM

RSM was applied to analyze the results of CIPF photodegradation utilizing 5% Co–ZnO NPs to develop an empirical model. These results led to the second-order polynomial equation (Eq. 18) being able to establish a connection between the parameters of the independent variables and the CIPF degradation.

18 $$ \begin{gathered} R = { 89}.{11} + .{\text{94A}} + {6}.{\text{96B }} + { 1}.0{\text{2C}} - {2}.{\text{81D}} - 0.{1}0{\text{86AB}} - {2}.{\text{75AC}} + {1}.{\text{46AD}} \hfill \\ \quad \quad - 0.{\text{7448BC}} - {1}.{\text{23BD}} + 0.{\text{9868CD}} + {2}.{\text{69A}}^{{2}} - {1}.{\text{55B}}^{{2}} + {1}.{\text{31C}}^{{2}} - {9}.{\text{24D}}^{{2}} \hfill \\ \end{gathered} $$

where *R* is response degradation percent, A, B, C, and D are the corresponding independent parameters such as catalyst dosage (g/L), antibiotic dosage (ppm), shaking speed (rpm), and pH. Using 5% Co–ZnO NPs as the catalyst, RSM was performed to determine the greatest CIPF removal by the photocatalysis processes as well as the ideal value for the variables such as initial antibiotic concentration, catalyst amount, shaking speed, and pH. The removal of the CIPF antibiotic was adjusted using experiments based on the central composite design and the second-order model. The statistical evaluation of the model was conducted using analysis of variance (ANOVA). Table 4 lists the coded and actual values for the initial concentration of the CIPF, the concentration of the 5% Co–ZnO NPs catalyst, the shaking speed, and the pH of the experimental design for the Co/ZnO for the 30 experiments. ANOVA estimation and expression of the correlation coefficient (R^2^) were used to fit the model. Table S4 presents the ANOVA findings for the fitted quadratic model of CIPF removal effectiveness as a function of the variables and parameter estimates.

**Table 4:** Experimental design result of CCD.

Std	Run	A Catalyst dosage	B Antibiotic dosage	C Shaking speed	D pH	Actual value	Removal predicted value	Residual
22	1	60	30	250	7	89.61	96.42	− 6.81
18	2	100	30	150	7	99.67	103.75	− 4.08
15	3	40	40	200	9	84.93	85.95	− 1.02
27	4	60	30	150	7	89.11	89.11	0.0000
6	5	80	20	200	5	74.23	75.56	− 1.33
4	6	80	40	100	5	90.69	97.15	− 6.46
14	7	80	20	200	9	80.29	77.29	2.99
2	8	80	20	100	5	82.92	79.50	3.42
24	9	60	30	150	11	33.40	46.51	− 13.11
29	10	60	30	150	7	89.11	89.11	0.0000
30	11	60	30	150	7	89.11	89.11	0.0000
16	12	80	40	200	9	90.44	87.04	3.40
5	13	40	20	200	5	79.39	79.88	− 0.4907
3	14	40	40	100	5	90.29	90.89	− 0.5996
11	15	40	40	100	9	87.34	77.92	9.42
26	16	60	30	150	7	89.11	89.11	0.0000
7	17	40	40	200	5	95.16	94.98	0.1776
20	18	60	50	150	7	95.23	96.81	− 1.58
25	19	60	30	150	7	89.11	89.11	0.0000
17	20	20	30	150	7	89.57	95.98	− 6.40
21	21	60	30	50	7	88.65	92.32	− 3.68
10	22	80	20	100	9	85.20	77.29	7.91
28	23	60	30	150	7	89.11	89.11	0.0000
19	24	60	10	150	7	60.08	68.99	− 8.91
9	25	40	20	100	9	61.32	64.76	− 3.44
1	26	40	20	100	5	77.51	72.82	4.69
12	27	80	40	100	9	92.90	90.02	2.88
8	28	80	40	200	5	96.05	90.22	5.83
23	29	60	30	150	3	60.38	57.75	2.62
13	30	40	20	200	9	90.32	75.77	14.55

As can be seen from the table, the second-order quadratic model's fit to the data was deemed significant because its F-value of 5.54 suggests that the model is significant and its P-values of less than 0.05 suggest that model terms are significant. The precision of the model is determined by the determination coefficient (*R*^2^). There is a correlation between the experimental data and expected values, as indicated by the correlation coefficient value (*R*^2^ = 0.8378)^73^. This showed that the independent variables could account for 83.7% of the variation in CIPF antibiotic elimination while the model could not account for 16.3% of the total variation. The adj-R^2^ value was discovered to be 0.68651, and it was determined that this value was compatible with the R^2^ value^73^. The experiments were more accurate and dependable because of the comparatively low coefficient of variation for the model (CV = 9.23%) for CIPF antibiotic elimination. The signal is sufficient as evidenced by the suitable precision value of 10.4828.

The model's predicted values and the actual results of the exam of the CIPF removal efficiency can be seen in Fig. S4a. The points provided in this system were reasonably close to the straight line and had excellent relationships, as shown by the results in Fig. S4. The residuals versus normal probability plots for CIPF degradation are shown in Fig. S4b. The acquired data display a linear connection, as shown in the plot, which suggests that the residuals are distributed normally. It follows that the parameterization of the CIPF deterioration using the established quadratic model provides reasonable predictions of the experimental results.

The perturbation plot, which is depicted in Fig. S5, was created to investigate the simultaneous impact of four variables on antibiotic elimination. Typically, these graphic contrasts the effects of all components at a specific location in the design space^74^. The variables that controlled the degradation of ciprofloxacin were the dosage of the catalyst (A), the dye concentration (B), the shaking speed (C), and the pH level (D). Therefore, the fact that the curve for antibiotic dosage exists shows that one element has a greater impact than the others.

**Figure 20:**
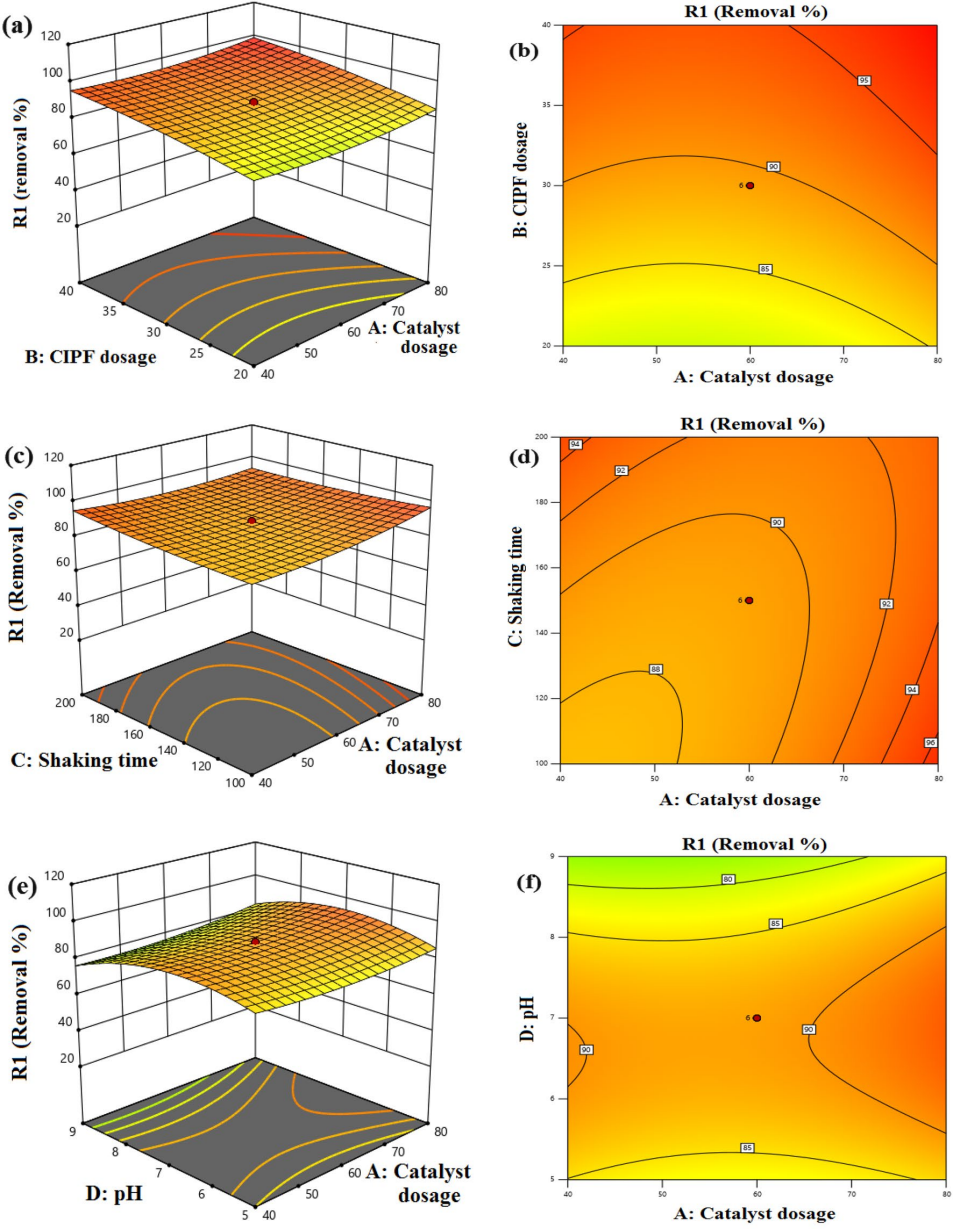

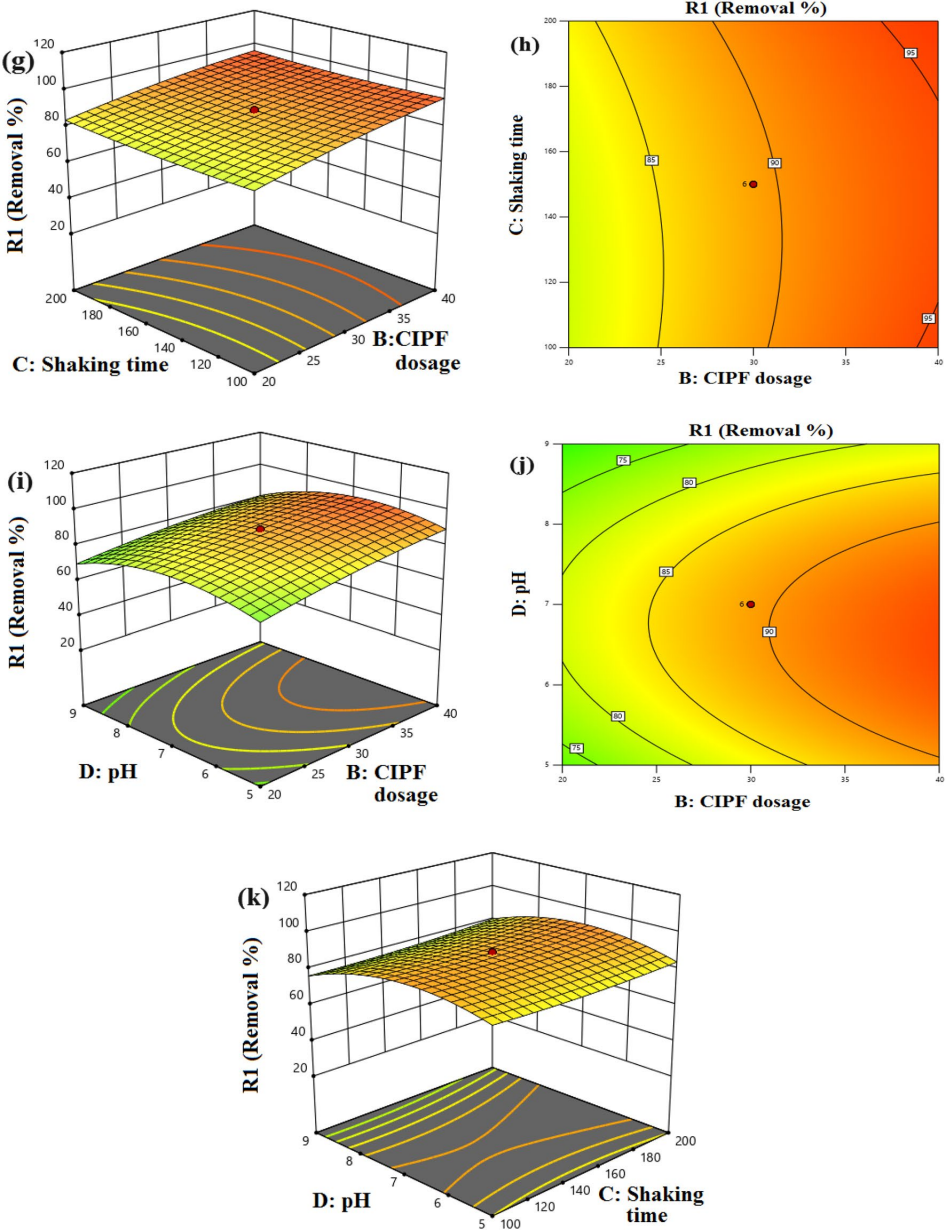
3D and 2D response surface plots, (**a**–**b**) CIPF dosage and Green 5% Co–ZnO NPs dosage, (**c**–**d**) shaking speed (rpm) and Green 5% Co–ZnO NPs dosage, (**e**–**f**) pH value and Green 5% Co–ZnO NPs dosage, (**g**–**h**) CIPF dosage and shaking speed, (**i**–**j**) pH value and CIPF dosage, (**k**–**l**) pH value and shaking speed.

A statistically sound model is used to create response surface curves, which offer ideal conditions and illustrate how different process elements interact. The regression equation is typically graphically represented by the 3D and 2D curves. Each curve suggests an infinite number of possible combinations of the other two variables while one variable remains constant. The interaction between the process variables is also shown by the 3D and 2D curves in Fig. 20a–l. Figure 20a, b shows the interaction impact of catalyst dose and CIPF initial concentration. The degradation of CIPF was shown to rise as the dosage was increased from 0.4 to 0.8 g/L, which had a substantial impact on the photocatalytic process. There are now more active sites available on the catalyst surface, which has improved reactivity with CIPF^75^, thereby enhancing the photocatalytic efficiency. The changes in the D.E.% of CIPF with photocatalyst amount and speed shaking are also shown in Fig. 20c, d. These numbers show that both variables positively affect the photocatalytic degradation efficiency of CIPF. From this, it can be concluded that the highest D.E.% can be obtained at the highest levels of these two elements. Figure 20e, f illustrates how the amount of photocatalyst, and the initial pH of the solution affect the photocatalytic degradation efficiency of CIPF. This graph makes it evident that the amount of photocatalyst has a critical impact on the D.E.% of CIPF and that this impact is continuous with rising photocatalyst amount. D.E.% approaches a maximum when pH 7 is applied, like the preceding figure. Figure 20g, h. illustrates the effects of CIPF initial concentration and shaking rate. The elimination of antibiotics increased as the shaking speed was increased. The interaction between pH and antibiotic concentration in aqueous solution demonstrates a positive trend up to pH 7 and a negative trend from pH 7 to 9, as shown in Fig. 20i, j. The effects of the solution's initial pH and shaking speed are shown in Fig. 20k, l. The clear effect of this parameter is seen by the fast increase in the D.E.% of CIPF with an increase in shaking speed. Additionally, it is evident that as pH is raised to around 7, the efficiency of degradation first rises and then falls.

**Figure 21:**
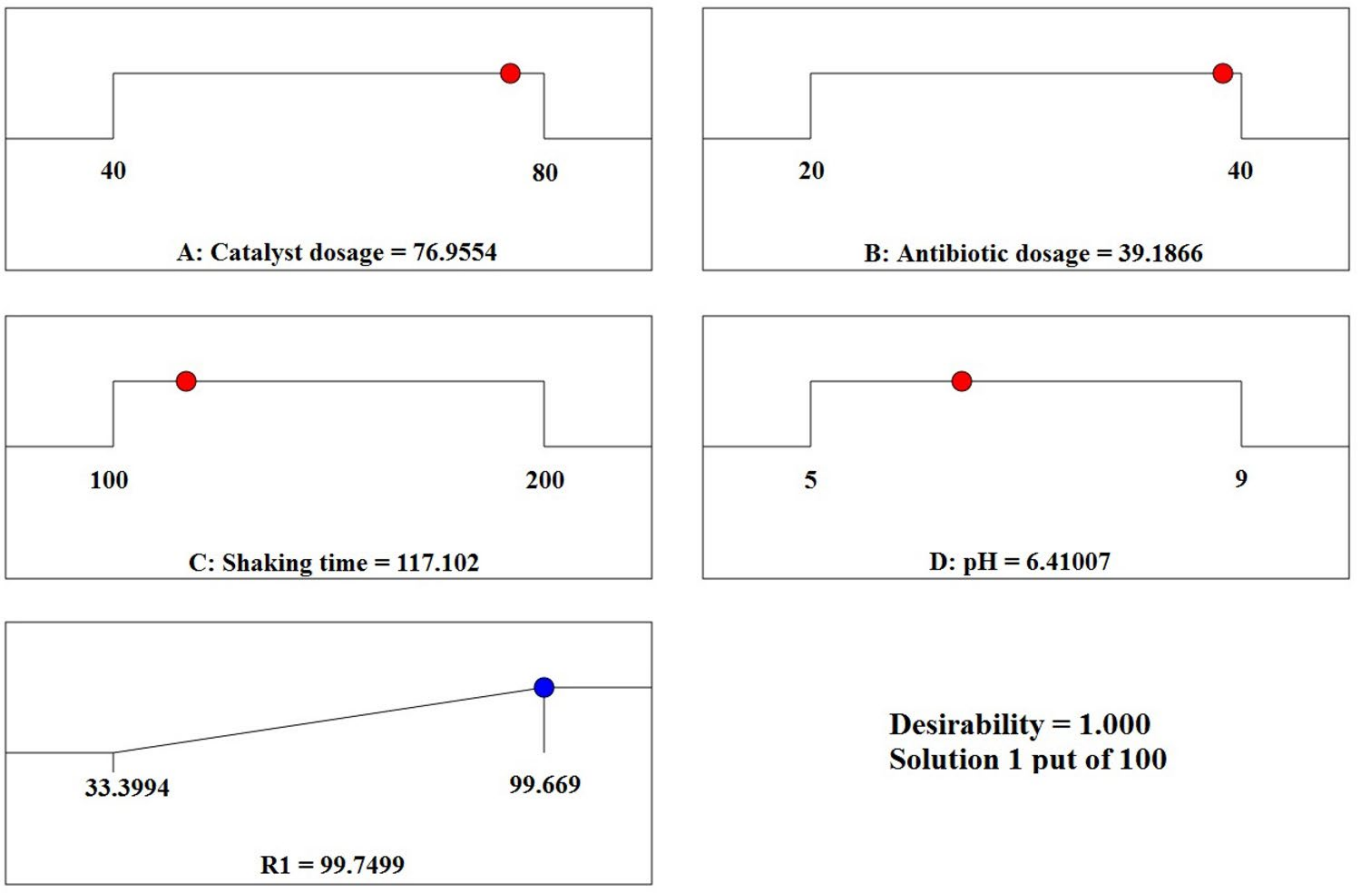
A final answer through CCD with optimized settings.

Derringer's desirability function was applied to achieve the highest removal efficiency of CIPF by 5% Co–ZnO NPs photocatalytic process under optimal circumstances. A number between 0 and 1, where 1 denotes the desired performance and 0 denotes the unwanted performance, was used in this function to determine the process function^76^. Favorable circumstances for the elimination of CIPF are shown in Fig. 21. The optimal CIPF initial concentration, 5% Co–ZnO NPs dosage, shaking speed, and pH values for the model were 39.19 mg/L, 76.96 mg, 117.1 rpm, and 6.41, respectively. In ideal circumstances, the model's estimated efficiency was 99.75%.

## Conclusions

The present study investigates the fabrication and characterization of Green ZnO NPs, 5, 10 and 15% Co–ZnO NPs using *P. Capillacea* extract for the CIPF antibiotic photodegradation. The using of *P. Capillacea* Algae extract because it contains a variety of biomolecules, such as proteins, peptides, carbohydrates, and pigments. These biomolecules can act as reducing agents, stabilizing agents, or capping agents during the synthesis of Co–ZnO Nps. They can facilitate the formation and growth of nanoparticles, control their size and shape, and prevent aggregation. The fabrication of Co–ZnO NPs was confirmed by FTIR, XRD, SEM, EDX, XPS, and DR-UV–Vis spectroscopy. The investigation of change in the lattice strain (ε) that arises due to the addition of Co to ZnO was confirmed by XRD. Moreover, the increase in the lattice strain on doping is associated with the increase in dislocation density. The photocatalytic degradation of CIPF showed that 5% Co–ZnO NPs significantly enhanced the photocatalytic activity with high removal reaching 100% after the first 15 min. The impact of each experimental parameter including recycling of 5% Co–ZnO NPs catalyst was studied. 5% Co–ZnO NPs dosage, pH, temperature, and CIPF concentrations on photocatalytic activity were studied as well and the reaction kinetics study was also studied. The results showed that first order is the suitable model to describe the reaction. Moreover, CCD optimization of 5% Co–ZnO NPs was also studied, and the result revealed that at Green 5% Co–ZnO NPs dosage of 76.96 mg, 39.19 mg/L of CIPF initial concentration, pH 6.41 and 117.1 rpm; the removal reached 99.75% after 45 min.

### Supplementary Information

Supplementary Information.

## Data Availability

Upon request to the corresponding author of the study, the datasets utilized in this inquiry are available for inspection.
