# Supplementary material for: Cube-shaped Cobalt-doped zinc oxide nanoparticles with increased visible-light-driven photocatalytic activity achieved by green co-precipitation synthesis
Source: Sci Rep. 2023 Nov 7;13:19329. doi: 10.1038/s41598-023-46464-7 (PMC10630306; doi:10.1038/s41598-023-46464-7)
Supplement: Supplementary file 1 — Supplementary Information. [file 41598_2023_46464_MOESM1_ESM.docx]

**Supplementary data**

**Cube-shaped Cobalt-doped zinc oxide nanoparticles with increased visible-light-driven photocatalytic activity achieved by green co-precipitation synthesis**

Asmaa I. Meky^1^, Mohamed A. Hassaan^2^, Howida A. Fetouh^1^, Amel M. Ismail^1^, Ahmed El Nemr^2^*

| 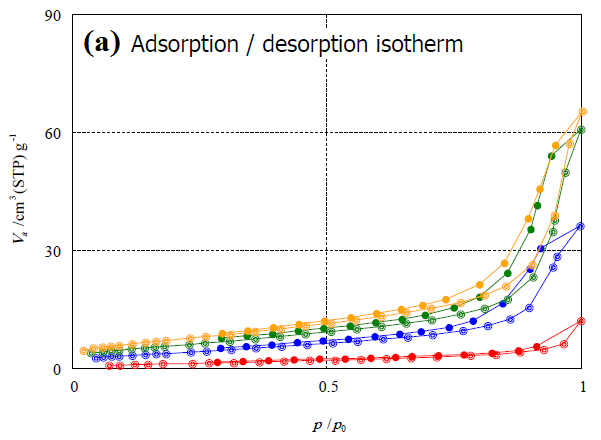 | 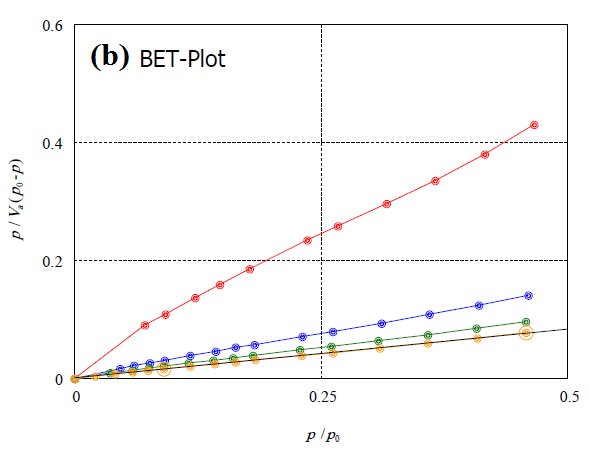 |
| --- | --- |
| 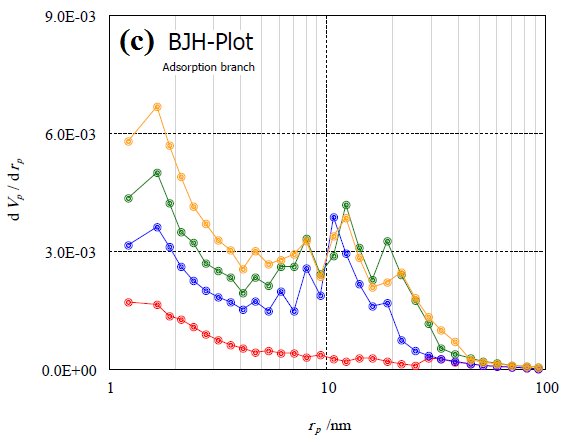 | |

**Figure S1.** (**a**) Adsorption–desorption of ZnO (red), 5%Co-ZnO (blue) ,10%Co-ZnO(green) and 15%Co-ZnO (yellow); (**b**) BET analys is; (**c**) BJH analysis by adsorption.

**Table S1**. Analysis of the surface area of Green ZnO NPs, 5, 10, and 15% Co-ZnO NPs.

| Model | Parameter | ZnO | 5% Co-ZnO | 10% Co- ZnO | 15% Co- ZnO |
| --- | --- | --- | --- | --- | --- |
| BET | *a*_s, BET_ (m^2^∕g) | 5.0310E+00 | 1.5114E+01 | 2.1985E+01 | 2.7207E+01 |
|  | *V*_m_ (cm^3^ STP)/g) | 1.1559 | 3.4726 | 5.0512 | 6.2509 |
|  | Mean pore diameter *P*_m_ (nm) | 13.71 | 14.356 | 16.330 | 14.035 |
|  | Volume of total pore *V*_T_ (cm^3^/g) | 1.6560E-02 | 5.42245E-02 | 8.9757E-02 | 9.5460E-02 |
| BJH ads | *V*_p_ (cm^3^/g) | 1.6727E-02 | 5.4121E-02 | 8.93356E-02 | 9.4243E-02 |
|  | *a*_p_ (m^2^/g) | 5.4328 | 15.557 | 22.152 | 26.212 |

**
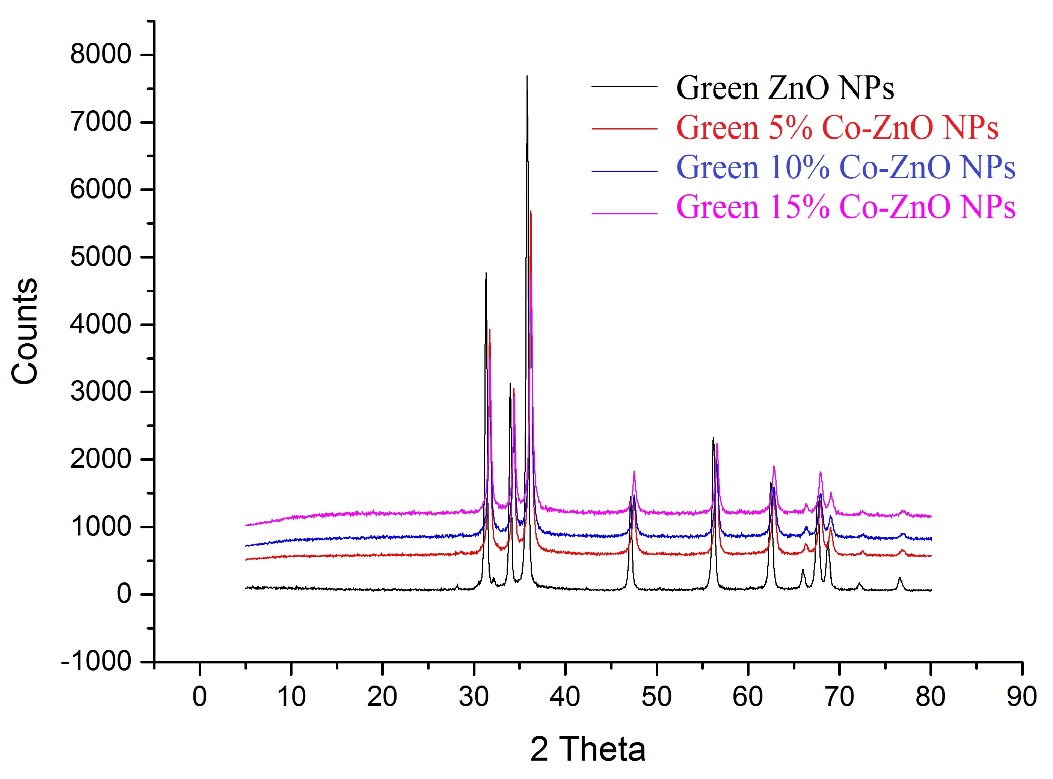
**

**Figure S2**. X-ray diffraction pattern of Green ZnO NPs, 5, 10, and 15% of Co-ZnO NPs.

**Table S2**. Crystal size of ZnO and 5, 10, 15% Co doped ZnO (nm)

| 2Theta | ZnO NPs | 2Theta | 5% Co-ZnO | 2Theta | 10% Co-ZnO | 2Theta | 15% Co-ZnO |
| --- | --- | --- | --- | --- | --- | --- | --- |
| 31.314 | 49.62 | 31.73 | 53.03 | 31.727 | 44.09 | 31.75 | 45.63 |
| 33.966 | 50.27 | 34.376 | 51.31 | 34.385 | 43.42 | 34.405 | 47.04 |
| 35.808 | 47.79 | 36.208 | 46.17 | 36.216 | 39.12 | 36.237 | 41.15 |
| 47.123 | 44.76 | 47.484 | 40.83 | 47.506 | 34.95 | 47.523 | 40.23 |
| 56.191 | 44.80 | 56.547 | 42.86 | 56.55 | 40.24 | 56.574 | 41.21 |
| 62.475 | 42.65 | 62.806 | 39.84 | 62.851 | 36.56 | 62.822 | 37.48 |
| 65.998 | 47.12 | 66.305 | 49.82 | 66.385 | 60.54 | 66.33 | 56.36 |
| 67.583 | 41.48 | 67.879 | 42.13 | 67.922 | 37.30 | 67.916 | 38.40 |
| 68.719 | 43.75 | 69.018 | 45.35 | 69.008 | 35.10 | 69.075 | 43.85 |
| 72.174 | 51.04 | 72.523 | 55.40 | 72.619 | 54.46 | 72.521 | 58.15 |
| 76.618 | 42.95 | 76.839 | 37.20 | 76.946 | 45.27 | 76.965 | 33.96 |

**Table S3.** Calculated structural and optical parameters for prepared samples.

| Sample | a (nm) | c (nm) | Crystallite  size (D) (nm) | disclosion density δ (10^-3^) | Microstrain ε (10^-3^) | Volume of unit cell (nm^3^) |
| --- | --- | --- | --- | --- | --- | --- |
| ZnO | 0.240 | 0.9074 | 47.79 | 0.438 | 2.242 | 0.04528 |
| Co-ZnO 5% | 0.2432 | 0.9112 | 46.17 | 0.469 | 2.296 | 0.04665 |
| Co-ZnO 10% | 0.2431 | 0.9123 | 39.12 | 0.653 | 2.709 | 0.04670 |
| Co-ZnO 15% | 0.2433 | 0.9112 | 41.15 | 0.590 | 2.574 | 0.04671 |

| 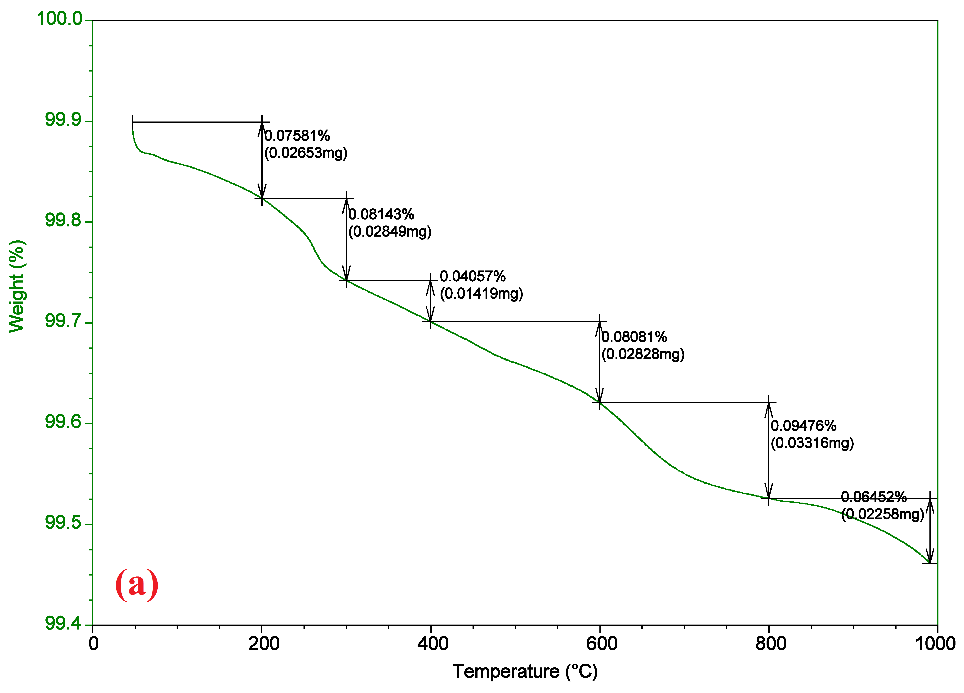 |
| --- |
| 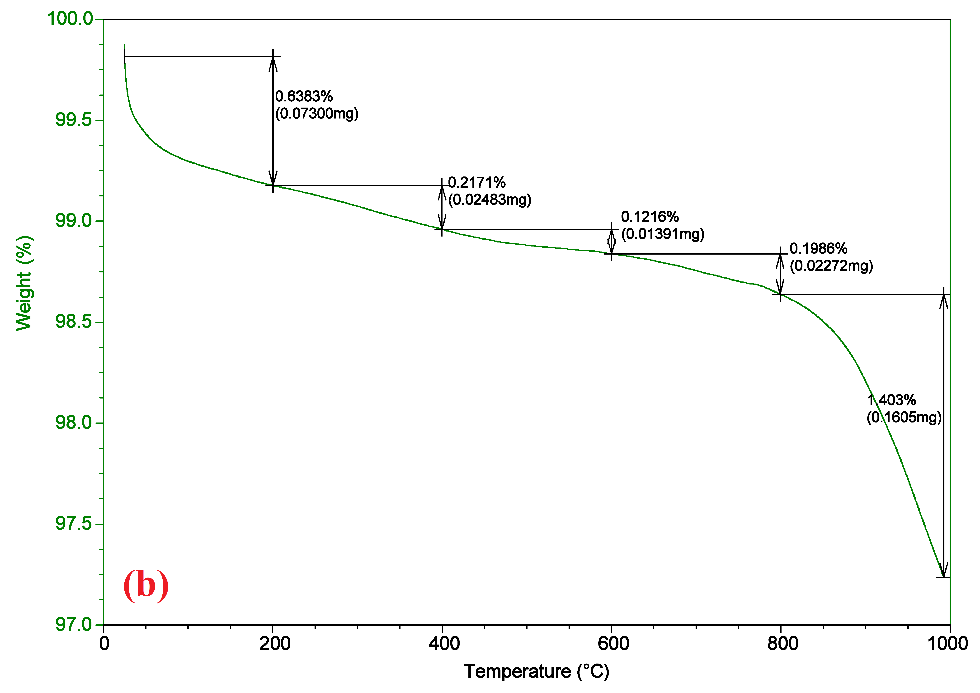 |
| 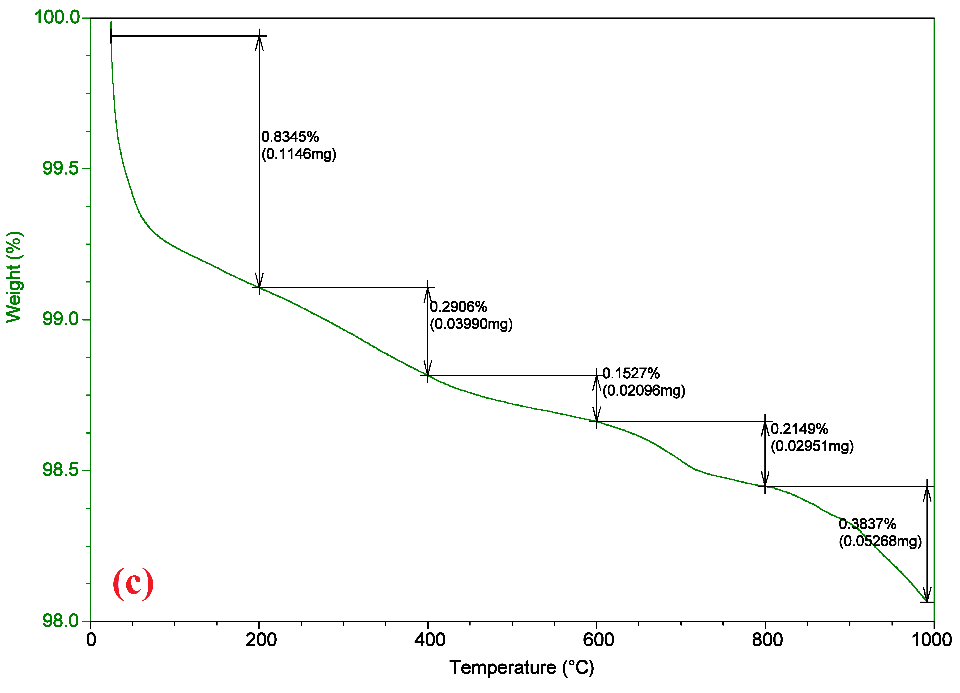 |
| 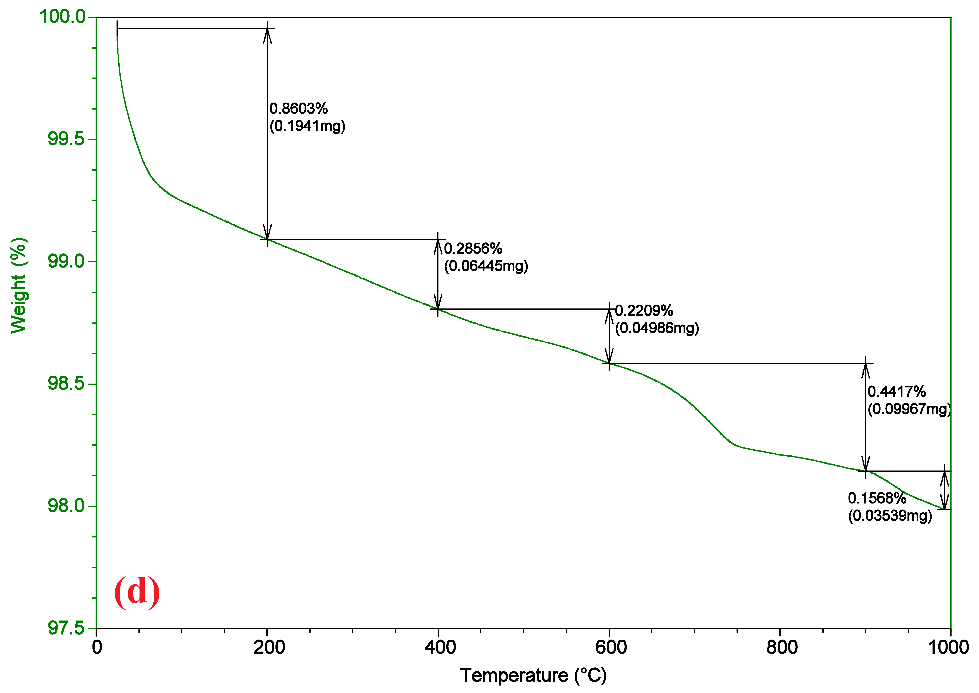 |

**Figure S3**. TGA analyses of (a) Green ZnO NPs, (b) Green 5% Co-ZnO NPs, (c) Green 10% Co-ZnO NPs, (d) Green 15% Co-ZnO NPs.

**Table S4**. A summary of the tested statistical method for CIPF degradation.

| **Source** | **Sum of Squares** | **df** | **Mean Square** | **F-value** | **p-value** |  |
| --- | --- | --- | --- | --- | --- | --- |
| **Model** | 4621.67 | 14 | 330.12 | 5.54 | 0.0011 | significant |
| A-Catalyst dosage | 90.70 | 1 | 90.70 | 1.52 | 0.2365 |  |
| B-Antibiotic dosage | 1161.09 | 1 | 1161.09 | 19.47 | 0.0005 |  |
| C- Shaking time | 25.14 | 1 | 25.14 | 0.4215 | 0.5260 |  |
| D-pH | 189.63 | 1 | 189.63 | 3.18 | 0.0948 |  |
| AB | 0.1888 | 1 | 0.1888 | 0.0032 | 0.9559 |  |
| AC | 121.23 | 1 | 121.23 | 2.03 | 0.1744 |  |
| AD | 34.18 | 1 | 34.18 | 0.5732 | 0.4607 |  |
| BC | 8.88 | 1 | 8.88 | 0.1488 | 0.7051 |  |
| BD | 24.16 | 1 | 24.16 | 0.4052 | 0.5340 |  |
| CD | 15.58 | 1 | 15.58 | 0.2613 | 0.6167 |  |
| A² | 198.25 | 1 | 198.25 | 3.32 | 0.0883 |  |
| B² | 66.12 | 1 | 66.12 | 1.11 | 0.3090 |  |
| C² | 47.41 | 1 | 47.41 | 0.7950 | 0.3867 |  |
| D² | 2344.16 | 1 | 2344.16 | 39.31 | < 0.0001 |  |
| **Residual** | 894.55 | 15 | 59.64 |  |  |  |
| Lack of Fit | 894.55 | 10 | 89.46 |  |  |  |
| Pure Error | 0.0000 | 5 | 0.0000 |  |  |  |
| **Cor Total** | 5516.22 | 29 |  |  |  |  |
| **R²** |  |  |  |  |  | 0.8378 |
| **Adjusted R²** |  |  |  |  |  | 0.6865 |
| **Predicted R²** |  |  |  |  |  | 0.0659 |
| **Adeq Precision** |  |  |  |  |  | 10.4828 |

| 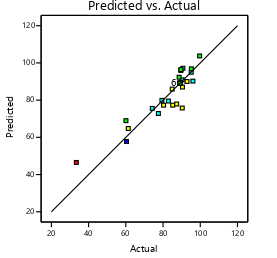 | 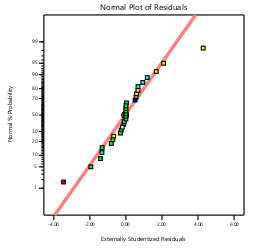 |
| --- | --- |
| **(a)** | **(b)** |

**Figure S4**. a-A plot of the predicted versus the experimental degradation Efficiency and b-the normal probability of the raw residuals.


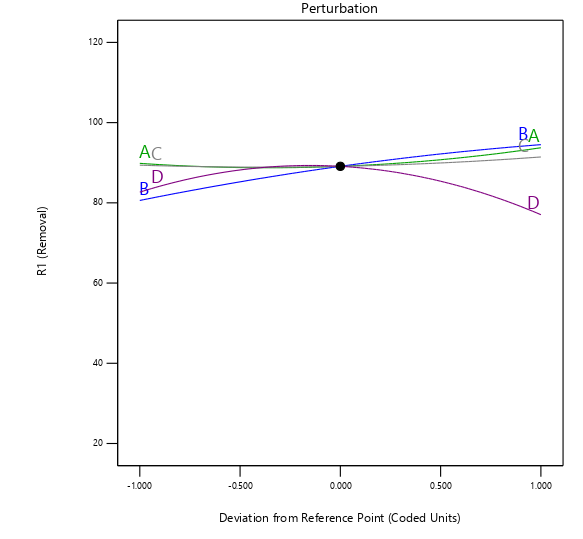


**Figure S5**. Perturbation plot showing the effect of each of the independent variables.
